# Histone H4 lysine 20 methylation marks genes dynamically regulated during erythroid maturation

**DOI:** 10.1186/s13072-025-00609-2

**Published:** 2025-07-26

**Authors:** Kristin Murphy, Michael Getman, Xiurui Lv, Zachary Murphy, Deanna Abid, Nabil Rahman, Michael Bulger, Laurie Steiner

**Affiliations:** 1 Department of Pediatrics, University of Rochester Medical Center, 601 Elmwood Ave, Box 703, Rochester, NY 14642 USA; 2 Center for RNA Biology, University of Rochester Medical Center, 601 Elmwood Ave, Box 703, Rochester, NY 14642 USA

**Keywords:** Histone methylation, H4K20me1, H4K20me3, Erythropoiesis, Differentiation

## Abstract

**Background:**

Methylation of H4K20 has been implicated in the regulation of gene expression but also plays essential roles in numerous cellular functions, making studies of its effects on transcription challenging. To gain insights into the role of H4K20 methylation in regulating gene expression, we studied H4K20me1 and H4K20me3 in the context of the well-characterized erythroid differentiation of human hematopoietic stem and progenitor cells.

**Results:**

H4K20me1 enrichment over the gene body was strongly correlated with expression changes. During erythroid differentiation, there was a dramatic decline in the level of RNA Polymerase II (Pol II); H4K20me1 was lost where Pol II was lost, and gained at genes where Pol II occupancy was maintained and transcripts were upregulated. We did identify a small subset of highly expressed genes, including beta-globin, that had a dramatic loss of H4K20me1 during erythroid differentiation, despite a substantial gain of Pol II. The histone demethylase PHF8 was present at these genes, as well as at the transcription start site of many active genes. In contrast to H4K20me1 over gene bodies correlating with transcription, enrichment at the transcription start site occurred at genes with low levels of Pol II occupancy and RNA expression throughout erythroid differentiation. The majority of H4K20me3 was present over intergenic regions, consistent with its well-established role as a repressor of repetitive elements. Intriguingly, H4K20me3 was also present at the transcription start site of genes with H4K20me1 over the gene body. At these genes, H4K20me3 levels were highly correlated with chromatin accessibility at the transcription start site, and an elevated Pol II pausing index. There was a dramatic loss of H4K20me3 occupancy in genic, but not intergenic, regions during erythroid differentiation.

**Conclusions:**

There are dynamic changes in H4K20 methylation during cellular differentiation that correlate strongly with Pol II occupancy and activity. These changes occurred in genic regions, with H4K20me3 at the transcription start site correlated with Pol II pausing, and H4K20me1 gene body levels tightly linked with transcriptional changes. Together, these data provide important insights into the role of H4K20 methylation in the regulation of gene expression during cellular differentiation.

**Supplementary Information:**

The online version contains supplementary material available at 10.1186/s13072-025-00609-2.

## Introduction

In eukaryotes, all DNA is packaged with histones to form chromatin. Posttranslational modifications of histones are essential for appropriate developmental- and celltype -specific patterns of gene expression, and also play roles in regulating key cellular functions such as cell cycle progression and DNA damage repair. Histone H4 Lysine 20 (H4K20) is evolutionarily conserved in eukaryotes from yeast to humans [1]. Methylation of H4K20 is important for multiple cellular processes, including cell cycle regulation [2, 3], DNA replication [4, 5], and DNA repair [6, 7], and interaction of H4K20 with the acidic patch of the histone H2A/H2B interface can affect higher order chromatin structure [8, 9]. In addition, methylation of H4K20 has been heavily implicated in the regulation of gene expression [10–12], but its essential roles in other cellular functions have presented challenges to investigation of its role in transcriptional regulation.

Setd8 (KMT5A) is the sole methyltransferase that mono-methylates H4K20. H4K20me1 is enriched over repetitive elements and actively transcribed gene bodies [13, 14], and has been implicated in both transcriptional activation and repression [8, 15–19]. H4K20me1 can be progressively methylated by KMT5B (SUV420H1) and KMT5C (SUV420H2) to H4K20me2 and H4K20me3 [16], although generation of H4K20me1 by KMT5A is required for subsequent progressive methylation to occur. The di-methylated form of H4K20 (H4K20me2) is present on ~ 80% of nucleosomes and has an important role in the coordination of DNA damage response, cell cycle progression, and DNA replication [20–22]. The tri-methylated and mono-methylated forms of H4K20 are present on approximately 5–10% of nucleosomes, although the abundance of all methylation states of H4K20 varies with the cell cycle, with H4K20me1 and H4K20me3 peaking during mitosis and G1/G0, and reaching their lowest levels during Sphase [6, 23]. H4K20me3 is enriched at telomeres and plays an important role in the repression of repetitive elements and maintenance of genome stability [24–27]. Studies in embryonic stem cells have identified H4K20me3 at bivalently marked promoters of developmentally regulated genes [25], and loss of KMT5C, the enzyme that tri-methylates H4K20, impairs embryonic stem cell differentiation [24]. H4K20me3 has also been implicated in the regulation of RNA Polymerase II (Pol II) pausing and higher order chromatin organization [24], but the role of H4K20me3 in regulating gene expression during cellular differentiation remains poorly understood.

Erythropoiesis is the process of making red blood cells from stem and progenitor cells. Human red blood cells have a limited life span of 120 days, and so the average adult produces approximately 2–3 million red cells per second to replace senescent cells and avoid the development of anemia [28]. The terminal maturation of red blood cell precursors is a well-characterized process that involves significant changes in gene expression, including the upregulation of erythroid genes such as globin and heme biosynthesis genes, as well as silencing of many non-erythroid genes [29]. These gene expression changes occur in the context of a nucleus that is rapidly condensing prior to enucleation. Setd8 has been shown to be essential for erythropoiesis, with loss of Setd8 resulting in cell cycle abnormalities, dysregulated gene expression, and defective nuclear condensation [11, 30]. As Setd8 plays an essential role in cell cycle progression and other essential cellular processes, delineating the effect of H4K20 methylation on erythroid gene expression has been challenging. In addition, genome-wide studies of H4K20me1 and H4K20me3 distribution are limited, particularly in primary human cells. In this study, we use the well-characterized erythroid differentiation of CD34+ hematopoietic stem and progenitor cells (HSPCs) to study changes in H4K20 methylation during cellular differentiation, and to study its association with Pol II and RNA expression. We find that H4K20 mono- and trimethylation are present at active genes that are dynamically regulated during terminal erythroid maturation, and strongly correlated with Pol II occupancy and activity. Further, we identified dramatic changes in H4K20me1, accompanied by H4K20me3 occupancy, at highly expressed genes, such as beta-globin. Together, these data provide important insights into the role of H4K20 methylation in the regulation of gene expression during cellular differentiation.

## Results

### Global changes in H4K20 methylation during erythroid maturation

**Fig. 1:**
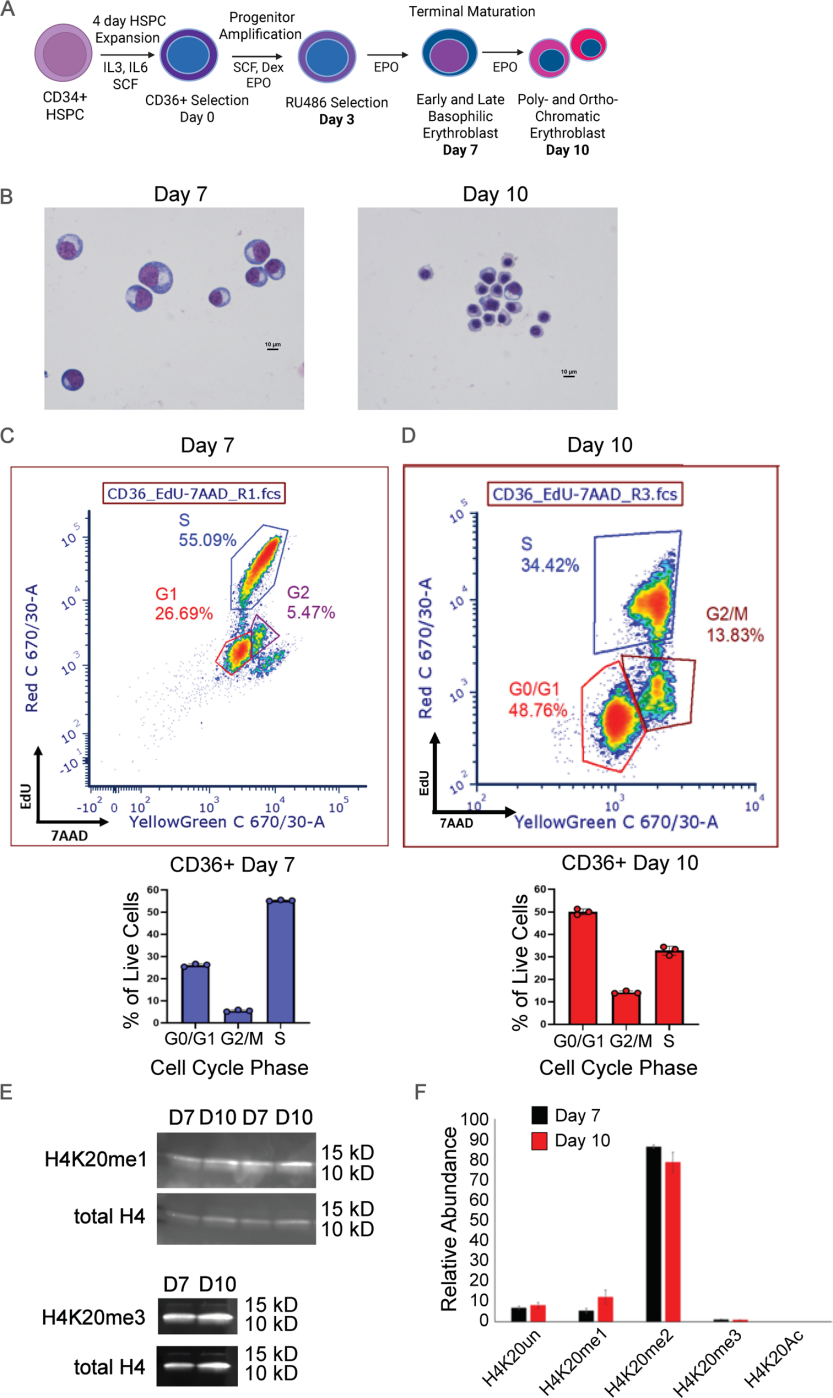
Figure 1. Global changes in H4K20 methylation during erythroid maturation. (A) Overview of semi-synchronous culture system, modified from Gautier 2016.31 (B) Cell morphology demonstrated by cytospin on days 7 and 10 in culture. (C-D) Cell cycle analyses determined by EdU incorporation at day 7 (C) and day 10 (D). Example flow cytometry plot (left) and percent of cells in each phase of the cell cycle (right). Data represent 3 independent replicates. (E)Western blot of H4K20 mono and tri methyl levels during the indicated culture days. (F) H4K20 modification status at day 7 and day 10 of culture as determined by mass spectrometry. Data from Murphy 2021.32 Data represent 2 independent replicates. Error bars represent standard error of the mean.

In this study, we set out to study the roles of H4K20me1 and H4K20me3 during normal erythroid differentiation. We isolated cells for study at day 7 and day 10 of the CD34 + derived erythroid culture and maturation system developed by Gautier et al. [31] and shown in Fig. 1A. At day 7 the cells are largely basophilic erythroblasts, while at day 10 they consist of poly- and ortho- chromatic erythroblasts which are still dividing and not yet enucleated, but have undergone significant nuclear condensation and transcriptional changes to support the functions of mature erythrocytes (Fig. 1B, Fig S1) [32]. 

Deposition of H4K20 methylation is tightly linked to the cell cycle, and terminal erythroid maturation consists of a limited number of cell divisions accompanied by phenotypically distinct cellular stages prior to cell cycle exit and enucleation. As the cells mature from basophilic erythroblasts to orthochromatic erythroblasts, they are also proliferating, with 55% of cells in S phase at day 7 (Fig. 1C). The percentage of cells in S-phase decreases to 35% at day 10 (Fig. 1D), as expected when the cells near enucleation. Despite the shift in the number of cells in S-phase, levels of H4K20me1 and H4K20me3 were relatively stable. Mass spectrometry demonstrated a modest increase in H4K20me1 (Fig. 1F, E, Fig S1B), although this change did not reach statistical significance. Thus, day 7 and day 10 cells are suitable for H4K20 methylation chromatin interrogation without cell cycle exit or enucleation confounding analyses.

### H4K20me1 is enriched over the bodies of highly transcribed genes

**Fig. 2:**
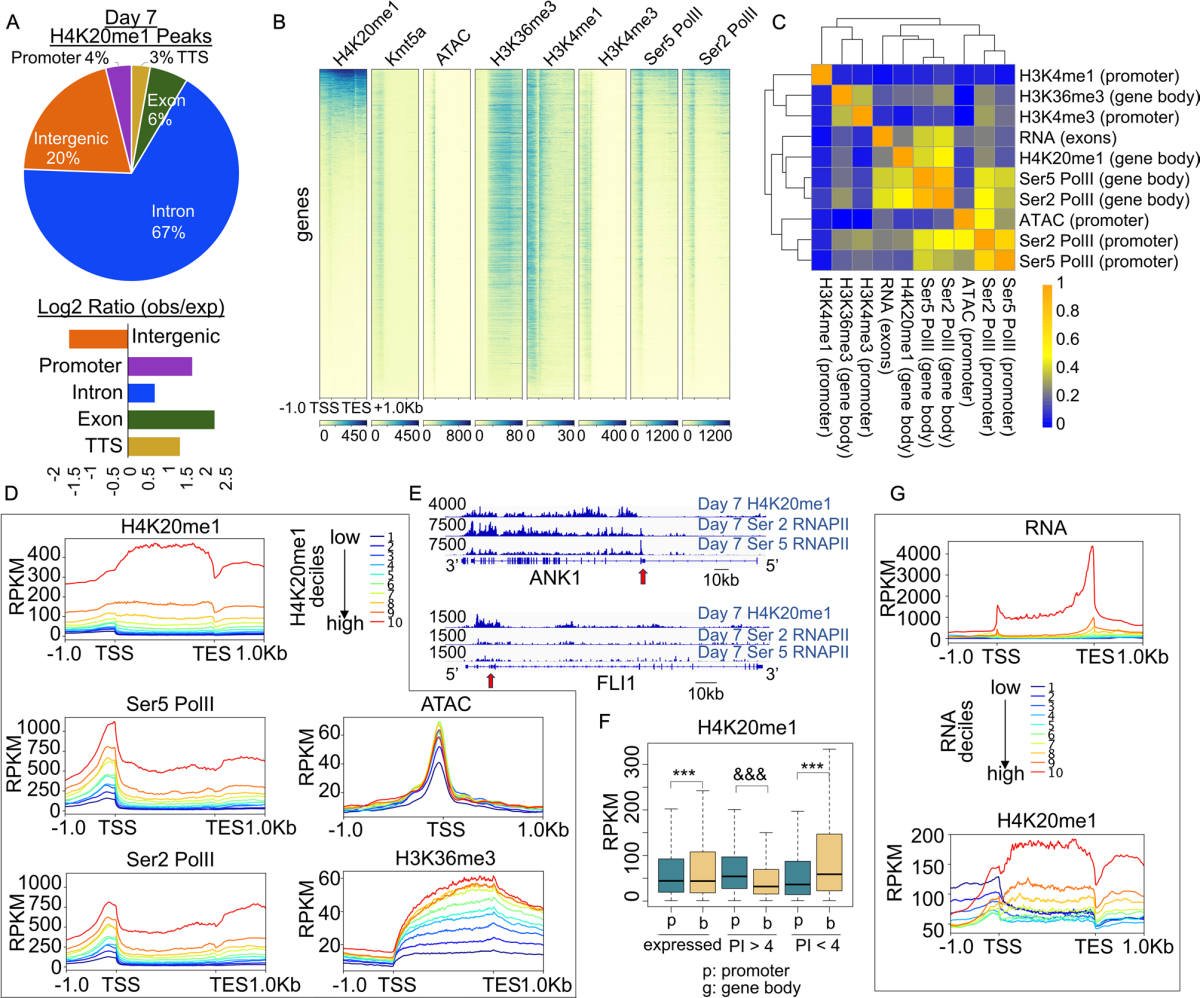
H4K20me1 is enriched over the body of active genes, and is highly correlated with RNA Polymerase II occupancy and RNA expression. (**A**) Genomic annotation of H4K20me1 peaks (total = 73,978) in day 7 erythroblasts using Homer annotatePeaks.pl, with associated log2 ratio of observed fraction of peaks assigned to each annotation compared to fraction of the annotated genome assigned to each annotation. (**B**) Heat map showing H4K20me1, KMT5A, chromatin accessibility (ATAC-seq), H3K36me3, H3K4me1, H3K4me3, serine 5 phosphorylated RNA Polymerase II (Ser5 Pol II), and serine 2 phosphorylated RNA Polymerase II (Ser2 Pol II) occupancy over genes sorted by H4K20me1 levels on day 7. (**C**) Day 7 Pearson correlation heatmap for average normalized scores of H4K20me1, H3K36me3, H3K4me3, H3K4me1, chromatin accessibility (ATAC), Ser5 Pol II, Ser2 Pol II, and RNA (RNA-seq) over promoters (TSS +/- 500 bp), gene bodies (TSS to TES), or merged exons for RNA (metagene) as indicated. (**D**) H4K20me1, Ser5 Pol II, Ser2 Pol II, H3K36me3, and chromatin accessibility plotted over genes divided into deciles based on ranked H4K20me1 levels (TSS to TES). (**E**) RNA and H4K20me1 plotted over genes divided into deciles based on ranked RNA levels (merged exons). (**F**) Boxplot showing day 7 average levels of H4K20me1 over promoters (TSS +/- 500 bp) or gene bodies (TSS to TES) for expressed genes (54,947), expressed genes with pausing index (PI) > 4 (23,747), and expressed genes with PI < 4 (31,200). *P* value represents Welch Two Sample t-test. *** indicates significantly increased, *p* value < 2.2e-16, &&& indicates significantly decreased, *p* value < 2.2e-16. (**G**) Gene tracks showing examples of H4K20me1, Ser5 Pol II, and Ser2 Pol II enrichment at day 7 over ANK1 and FLI1 genes, y axis represents RPKM, genome scale bar as indicated

To examine the distribution of H4K20 methylation across the genome, we applied the CUT&RUN assay to the erythroid primary cell culture system shown in Fig. 1A. We first examined the profile of H4K20me1 in erythroid cells at day 7, using both CUT&RUN [33] and CUT&Tag [34]. There was high correlation between the two datasets (Fig S2A), however the CUT&Tag dataset had slightly less background, so it was chosen for further analyses. At day 7, H4K20me1 was located primarily over gene bodies, but was also present at promoters (Fig. 2A). Interrogation of the chromatin landscape at regions of H4K20me1 occupancy revealed enrichment for KMT5A and H3K36me3, a histone modification associated with active transcription by RNA Polymerase II (Pol II) (Fig. 2B). Transcriptional elongation is a tightly regulated process whereby Pol II is released from a paused state downstream of the TSS (transcription start site) through phosphorylation of serine 5 (Ser5) and serine 2 (Ser2) residues of the Pol II C-terminal domain [35]. Levels of H4K20me1 were highly correlated with the levels of both Ser5 and Ser2 phosphorylated Pol II (Ser5 Pol II and Ser2 Pol II) (Fig. 2C, Fig S2B-C), consistent with previous studies [19]. We separated regions of H4K20me1 occupancy into deciles, with decile 1 having the lowest H4K20me1 enrichment and decile 10 having the highest enrichment over gene bodies. The deciles with the highest H4K20me1 enrichment also had the highest enrichment for H3K36me3, and for both serine 5- and serine 2-phosphorylated Pol II (Fig. 2D). Genes marked with H4K20me1 over the body also had accessible chromatin at their promoters, but the amount of H4K20me1 enrichment was not strongly correlated with the level of chromatin accessibility (Fig. 2C-D, Fig S2D). The highly expressed ankyrin-1 (Ank1) cytoskeleton gene essential for erythroid development, and lowly expressed Fli1 gene for which downregulation promotes erythroid differentiation, are exemplified with tight correlation of H4K20me1 and Pol II levels over their gene bodies (Fig. 2E, Fig S2G).

Since Pol II levels appeared to be tightly correlated with H4K20me1 enrichment, we next assessed the relationship between H4K20me1 occupancy and RNA levels as determined by RNA-seq. We separated genes into deciles by expression levels, with decile 1 having the lowest RNA expression and decile 10 having the highest. The deciles with the highest RNA expression also had the highest levels of H4K20me1 over the gene body and the highest level of KMT5A at the TSS (Fig. 2F, Fig S2E-H). Interestingly, the three deciles with the lowest RNA expression had broad regions of H4K20me1 over the transcription start site (TSS) with lower levels of H4K20me1 over the gene body, as seen for the Fli1 gene (Fig. 2E, F, Fig S2F), suggesting that H4K20me1 enrichment over the transcription start site may be associated with transcriptional repression.

H4K20me1 enrichment at the promoter has been associated with Pol II pausing. Pol II pausing is often measured by the pausing index (PI), which is defined as the ratio of Pol II occupancy at the promoter to occupancy over the gene body. A pausing index of > 4 is often used to identify highly paused genes [36]. Intriguingly, at genes with a PI > 4, H4K20me1 levels were higher at the transcription start site (TSS) than the gene body, while at genes with a PI < 4 H4K20me1 levels were lower at the TSS and more enriched over the gene body (Fig. 2G). These results further suggest that H4K20me1 enrichment over the gene body is highly correlated with Pol II occupancy and RNA levels, while H4K20me1 enrichment over the TSS may attenuate transcription.

### H4K20me3 is located at the promoters of active genes and is highly correlated with chromatin accessibility at the promoter

**Fig. 3:**
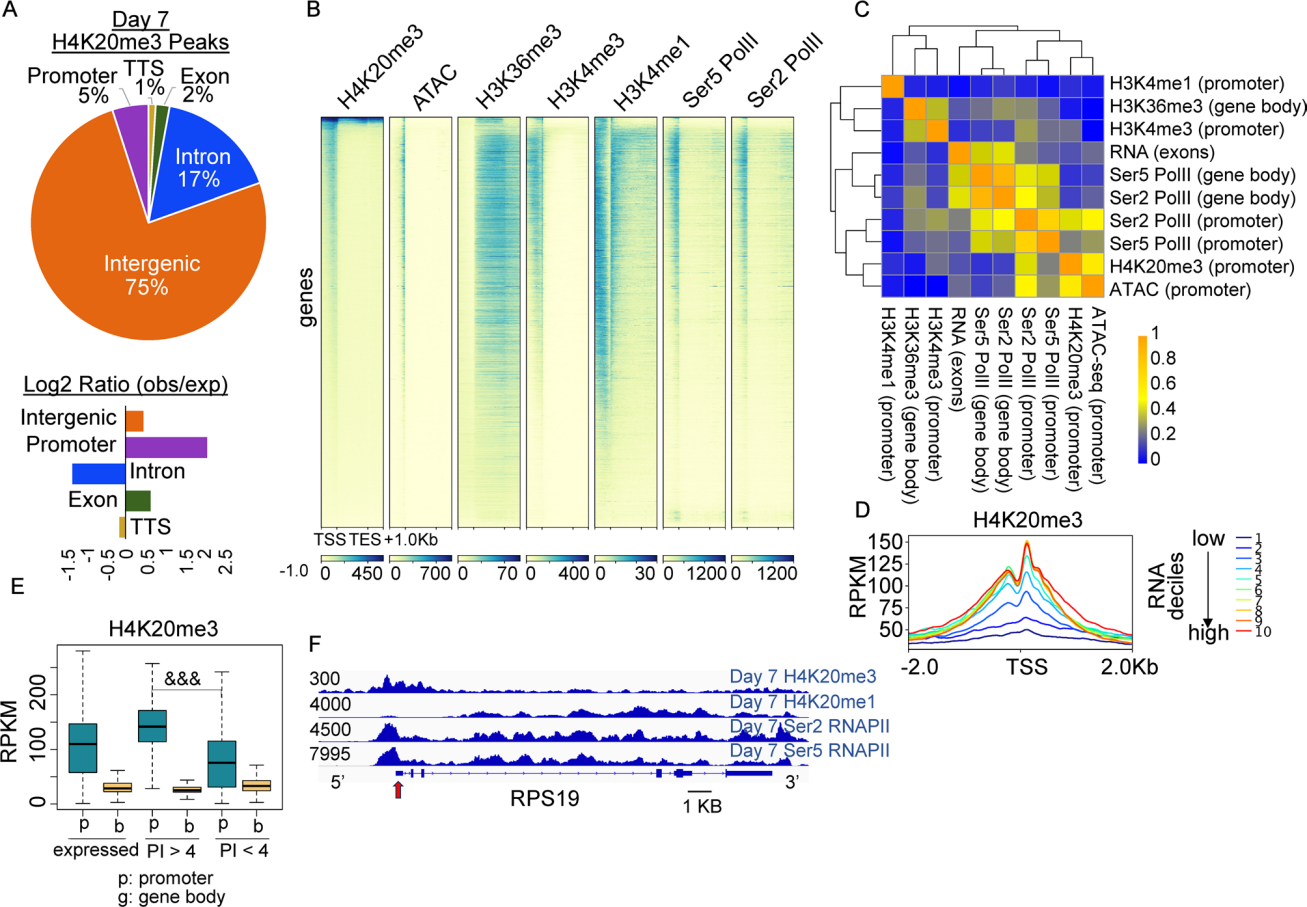
H4K20me3 is enriched at the TSS of active genes and is correlated with chromatin accessibility. (**A**) Genomic annotation of H4K20me3 peaks (total = 35,609) in day 7, using Homer annotatePeaks.pl with associated log2 ratio of observed fraction of peaks assigned to each annotation compared to fraction of the annotated genome assigned to each annotation. (**B**) Heat map showing H4K20me3, chromatin accessibility (ATAC-seq), H3K36me3, H3K4me3, H3K4me1, serine 5 phosphorylated RNA Polymerase II (Ser5 Pol II), and serine 2 phosphorylated RNA Polymerase II (Ser2 Pol II) occupancy over genes sorted by H4K20me3 levels on day 7. (**C**) Day 7 Pearson correlation heatmap for average normalized scores of H4K20me3, H3K36me3, H3K4me3, H3K4me1, chromatin accessibility (ATAC), Ser5 Pol II, Ser2 Pol II, and RNA (RNA-seq) over promoters (TSS +/- 500 bp), gene bodies (TSS to TES), or merged exons for RNA (metagene) as indicated. (**D**) H4K20me3 over genes (centered over TSS) divided into deciles based on ranked RNA levels (merged exons). (**E**) Boxplot showing day 7 average levels of H4K20me3 over promoters (TSS +/- 500 bp) or gene bodies (TSS to TES) for expressed genes (54,947), expressed genes with pausing index (PI) > 4 (23,747), and expressed genes with PI < 4 (31,200). *P* value represents Welch Two Sample t-test. &&& indicates significantly decreased, *p* value < 2.2e-16. (**F**) Gene tracks showing example of H4K20me3, H4K20me1, Ser5 Pol II, and Ser2 Pol II enrichment at day 7 over the RPS19 gene, y axis represents RPKM, genome scale bar as indicated

We next profiled H4K20me3 at day 7 of the erythroid culture system shown in Fig. 1A, with high correlation observed between CUT&RUN replicates (Fig. 3A). Consistent with its well-established role in the repression of repetitive elements [37, 38], H4K20me3 is present primarily at intergenic regions (Fig. 3A). Intriguingly, however, H4K20me3 was also enriched at genic regions, including promoters and to a lesser extent exons (Fig. 3A). Interrogation of the chromatin landscape at these genic regions revealed that H4K20me3 was present at the promoters of actively transcribed genes that were also marked with H3K4me3, H3K36me3, and Pol II (Fig. 3B-C, Fig S3B). H4K20me3 enrichment was highly correlated with the level of chromatin accessibility at the TSS (Fig. 3B-C, Fig S3B), but not as strongly correlated with levels of H3K36me3 or H4K20me1 over the gene body. While promoter H4K20me3 was enriched over genes expressing steady-state RNA (Fig. 3D, deciles 5–10), H4K20me3 levels did not correlate well with RNA expression levels (Fig. 3C-D, Fig S3D-F). As H4K20me3 has been associated with Pol II pausing, we next assessed the relationship between H4K20me3 levels and the PI. H4K20me3 levels at the TSS were significantly higher at highly paused genes (pausing index > 4), than at genes with lower levels of pausing (pausing index < 4; Fig. 3E). Together, these data demonstrate that H4K20me3 is enriched at the promoters of both transcriptionally active and paused genes occupied by RNA Pol II. An example of H4K20me3 and Pol II occupancy are shown at the ribosomal protein 40 S subunit gene RSP19 (Fig. 3F).

### Loss of H4K20me1 is correlated with loss of RNA polymerase II

**Fig. 4:**
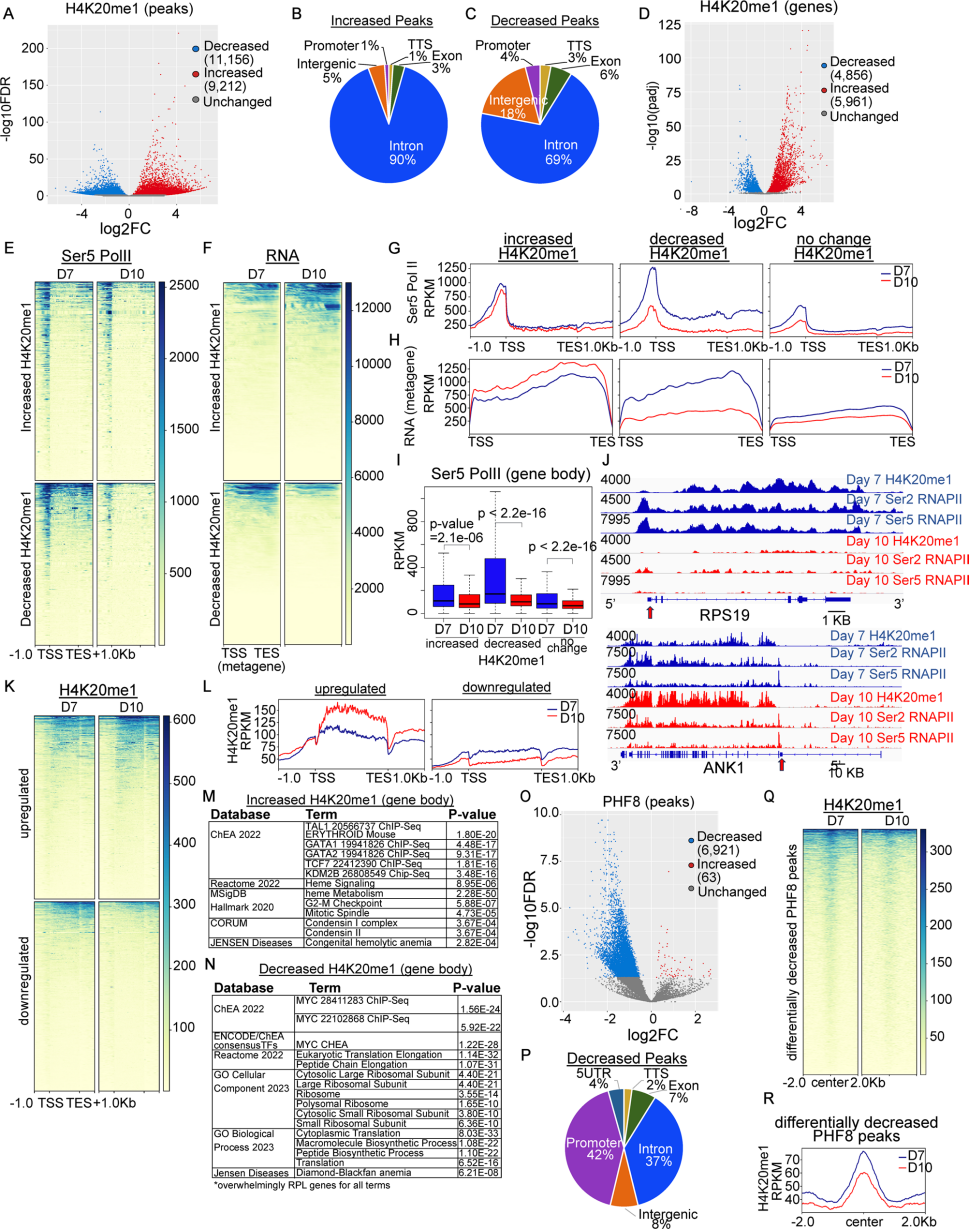
Loss of H4K20me1 is associated with loss of Pol II. (**A**) Volcano plot of differential H4K20me1 peaks during maturation, day 10 vs. day 7. (**B**-**C**) Genomic annotation of differentially increased (9,212) and decreased (11,158) H4K20me1 peaks. (**D**) Volcano plot of differential H4K20me1 over gene bodies (TSS to TES) during maturation, day 10 vs. day 7. (E-F) Heat map and (**G**-**H**) profile plot of serine 5 phosphorylated RNA Polymerase II (Ser5 Pol II) and RNA (merged exons) over genes that differentially gain and lose gene body H4K20me1 during maturation. (**I**) Boxplot showing average levels of Ser5 Pol II (TSS to TES) at day 7 (blue) and day 10 (red) over genes that gain and lose gene body H4K20me1 during maturation. (**J**) Gene tracks showing examples of H4K20me1, Ser5 Pol II, and Ser2 Pol II enrichment at day 7 (blue) and day 10 (red) over RPS19 and ANK1 genes, y axis represents RPKM, genome scale bar as indicated. (**K**) Heat map and (**L**) profile plot of H4K20me1 over differentially upregulated and downregulated genes, day 10 vs. day 7. (**M-N**) Gene ontology and pathway analysis for genes that differentially gain (**M**) or lose (**N**) H4K20me1 over gene bodies. (**O**) Volcano plot of differential PHF8 peaks during maturation, day 10 vs. day 7. (**P**) Genomic annotation of differentially decreased PHF8 peaks. (**Q**) Heat map and (**R**) profile plot of H4K20me1 over peaks that differentially lose PHF8, day 10 vs. day 7

We next examined changes in H4K20me1 occupancy that occur during erythroid maturation by comparing H4K20me1 CUT&Tag [34] performed at Day 7 and Day 10 of the cell culture system shown in Fig. 1A. Differential occupancy analyses at peaks identified regions both losing and gaining H4K20me1 (9,212 v 11,156, LFC +/- 1.5; Fig. 4A), although the magnitude of change was higher for regions that gained H4K20me1 peaks, and substantially higher when the analysis was limited to gene bodies (Fig. 4B-D, S4A). The regions that gained H4K20me1 during maturation were almost exclusively genic regions (~ 95%). In contrast, the regions that lost H4K20me1 had a genomic distribution similar to that of H4K20me1 on day 7, although the majority of these still occur within genes (Fig. 4B, C).

During the terminal maturation of erythroid progenitors, RNA Polymerase II (Pol II) levels decline dramatically, and the remaining Pol II is allocated to genes essential for the erythroid maturation process [32]. We found that changes in Pol II correlated with changes in H4K20me1 in genic regions; genes that gained H4K20me1 over the gene body largely maintained their levels of Pol II, accompanied by increased RNA (as shown for Ank1), while regions that lost H4K20me1 also exhibited a dramatic loss in Pol II occupancy and RNA (as shown for RPS19) (Fig. 4E-J, Fig S4B-D). Consistent with the changes in Pol II, H4K20me1 enrichment increased at upregulated genes and was lost at repressed genes (Fig. 4K-L). Pathway analyses of regions that gained H4K20me1 over the gene body revealed erythroid terms including heme signaling, and heme metabolism (Fig. 4M). In contrast, regions that lost H4K20me1 were enriched for terms related to ribosomes and translation (Fig. 4N). Together, these data demonstrate that dynamic changes in H4K20me1 over the gene body are associated with changes in both Pol II occupancy and RNA expression.

In contrast to H4K20me1 at the gene body, f genes with HK20me1 over the transcription start site at day 7 had low levels of Pol II occupancy, chromatin accessibility, and RNA expression at day 7, which remained low at day 10 (Fig. S4E-G). These data indicate that H4K20me1 at the TSS marks genes that are transcriptionally repressed prior to the onset of erythroid differentiation.

To understand factors that could mediate changes in H4K20 methylation, we interrogated the occupancy of the histone demethylase PHF8, which can demethylate H4K20me1 and H3K9me1 and positively regulates gene expression [39, 40]. We found that at day 7, PHF8 was present at promoters, introns, and intergenic regions (Fig S4H). PHF8 occupancy was correlated with chromatin accessibility, H4K20me3 and RNA levels (Fig S4I-L). Genes with PHF8 at the promoter exhibited low levels of H4K20me1 at the transcription start site but were enriched for H4K20me1 across the gene body, in addition to KMT5A, H3K4me1 and H3K4me3 at promoters (Fig S4M-O). During maturation, numerous regions lost PHF8 (Fig. 4O) and a large proportion of regions that lost PHF8 were located in promoters (Fig. 4P). Few sites gained PHF8, and they were not associated with any known pathways. Of note, PHF8 occupancy was maintained at genes expressed at late stages in erythropoiesis, including SLC4A1 (Band3), GYPA, and ANK1 (Fig S4P, Q). Regions that lost PHF8 also lost Pol II and were enriched for pathways silenced during erythropoiesis, including transcription, metabolism, and ribosomal related terms (Fig S4R-T). However, these regions did not gain H4K20me1 (Fig. 4Q-R). Taken together, these data suggest that while PHF8 may play a role in restricting H4K20me1 to gene bodies of expressed genes, it is not a major regulator of H4K20me1 changes during late stage erythropoiesis.

### H4K20me3 is enriched at genes that are dynamically regulated during erythroid maturation

**Fig. 5:**
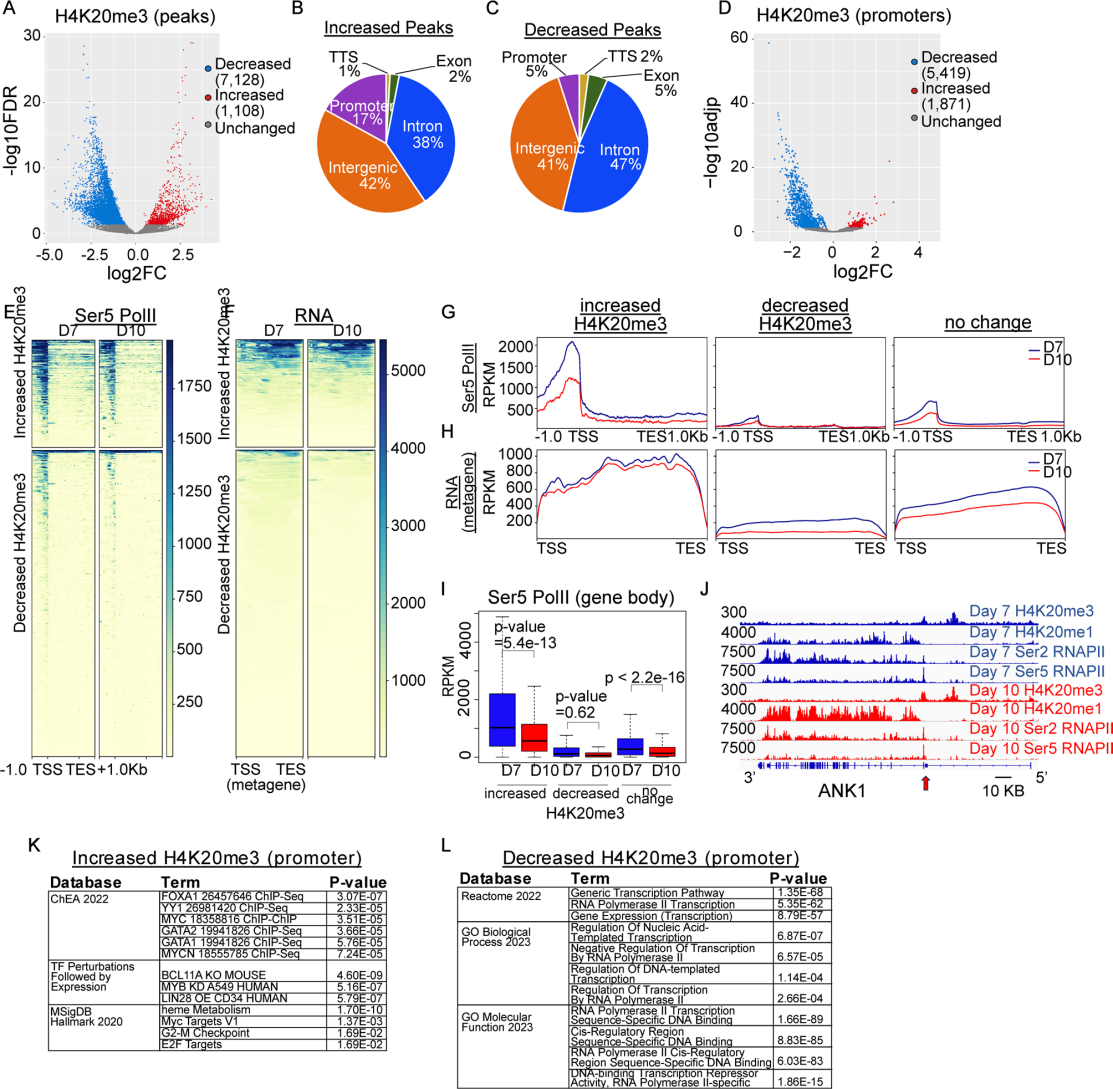
H4K20me3 is associated with dynamically regulated genes with a high pausing index. (**A**) Volcano plot of differential H4K20me3 peaks during maturation from day 7 to day 10. (**B**-**C**) Genomic annotation of differentially increased (1,108) and decreased (7,128) H4K20me3 peaks. (**D**) Volcano plot of differential H4K20me3 over promoters (TSS +/- 500 bp) during maturation from day 7 to day 10. (**E**-**F**) Heat map and (**G**-**H**) profile plot of serine 5 phosphorylated RNA Polymerase II (Ser5 Pol II) and RNA (merged exons) over genes that gain and lose promoter H4K20me3 during maturation. (**I**) Boxplot showing average levels of Ser5 Pol II (TSS to TES) over genes that gain and lose promoter H4K20me3 during maturation. (**J**) Gene tracks showing example of H4K20me3, H4K20me1, Ser5 Pol II, and Ser2 Pol II enrichment at day 7 (blue) and day 10 (red) over the ANK1 gene, y axis represents RPKM, genome scale bar as indicated. (**K-L**) Gene ontology and pathway analysis for genes that differentially gain (**K**) or lose (**L**) H4K20me3 over promoters

H4K20me3 also exhibited dynamic changes during terminal maturation. In contrast to H4K20me1, a substantially higher proportion (86%) of regions lost H4K20me3 at day 10 compared to day 7 (Fig. 5A). Although H4K20me3 was present largely at intergenic regions, dynamic changes in H4K20me3 occupancy occurred primarily in genic regions (Fig. 5B, C). We therefore interrogated changes in H4K20me3 specifically at promoters, noting that promoters exhibited a dramatic loss of H4K20me3 during erythroid maturation (Fig. 5D). Consistent with a role for H4K20me3 at active genes, regions that gained H4K20me3 during maturation showed robust enrichment for Pol II at both timepoints, while regions that lost H4K20me3 showed low levels of Pol II at day 7 that remained low at day 10 (Fig. 5E-J, S5A-C). Although both upregulated and downregulated genes were marked with H4K20me3 (Fig S5 D-E), regions that gained H4K20me3 had higher levels of RNA expression than regions that lost H4K20me3 (Fig. 5F, Fig S5F). These genes were enriched for erythroid-related terms, including heme metabolism (Fig. 5K), while promoters that lost H4K20me3 were enriched for terms related to Pol II (Fig. 5L). These data suggest H4K20me3 marks dynamically regulated, active genes in maturing erythroid cells.

### H4K20me1 and H4K20me3 maintain distinct regions of occupancy during erythroid maturation

**Fig. 6:**
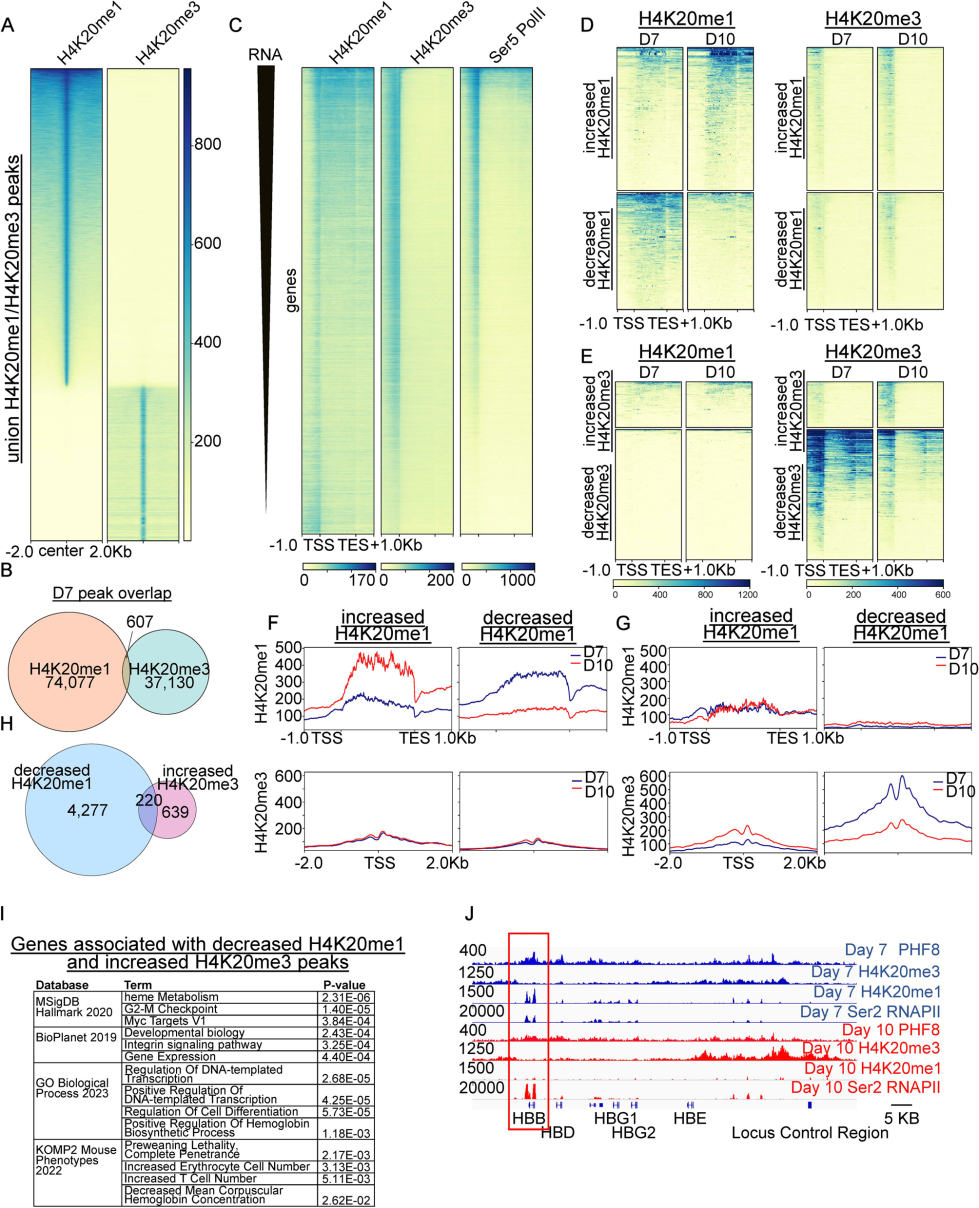
H4K20me1 and H4K20me3 are mutually exclusive in maturing erythroid cells, with few regions of progressive methylation. (**A**) Heatmap showing H4K20me1 and H4K20me3 occupancy over union H4K20me1/H4K20me3 peaks (sorted by H4K20me1 levels) at day 7 of maturation. (**B**) Venn diagram showing direct overlap in day 7 H4K20me1 and H4K20me3 peaks (**C**) Heatmap showing H4K20me1, H4K20me3, and serine 5 phosphorylated RNA Polymerase II (Ser5 Pol II) occupancy over genes sorted by average RNA (exons merged). (**D**-**E**) H4K20me1 and H4K20me3 heatmaps over genes with differentially increased and decreased H4K20me1 (**D**) or H4K20me3 (**E**). (**F**-**G**) Profile plots showing H4K20me1 (top) and H4K20me3 (bottom) over same regions as (**D**-**E**). (**H**) Venn diagram showing overlap in genes associated with decreased H4K20me1 peaks and increased H4K20me3 (**I**) Gene ontology and pathway analysis for shared set of genes (220) from (**H**) associated with decreased H4K20me1 peaks and increased H4K20me3. (**J**) Gene tracks showing example of PHF8, H4K20me3, H4K20me1, and Ser2 Pol II enrichment at day 7 (blue) and day 10 (red) over the beta-globin locus including locus control region, HBB boxed in red

At both day 7 and day 10, regions of enrichment (peaks) for H4K20me1 and H4K20me3 were mutually exclusive, with very few regions of direct overlap (Fig. 6A-B). Notably, however, a subset of genes enriched for Pol II had H4K20me3 at the promoter and were enriched for H4K20me1 over the gene body (Fig. 6C, Fig S6A-C, E). As H4K20me1 can be progressively methylated to H4K20me2 and then H4K20me3, we investigated the possibility that regions which lost H4K20me1 during maturation were being converted to H4K20me3. To determine if progressive methylation to H4K20me3 was accounting for changes in H4K20me1 occupancy during erythroid maturation, we assayed levels of H4K20me3 at regions that gained or lost occupancy of H4K20me1 in maturing erythroid cells (Fig. 6D-G). We identified surprisingly few regions that lost H4K20me1 and subsequently gained H4K20me3, suggesting that these marks maintain distinct regions of occupancy during erythroid maturation (Fig. 6D-G). The small number of regions that lost H4K20me1 and subsequently gained H4K20me3 were highly enriched for erythroid terms (Fig. 6H-I). These regions were highly enriched for PHF8 at both timepoints (Fig. 6I, S6D). Intriguingly, at the beta-globin locus, H4K20me1 was dramatically lost over the beta-globin gene but was gained over the locus control region, the enhancer that controls beta-globin expression (Fig. 6J), while maintaining H4K20me3. The highly expressed histone gene cluster containing H1/H2B/H3 also lost H4K20me1 during maturation, however, maintained enrichment for H4K20me3 (Fig S6D). H4K20me3 can also be demethylated to H4K20me2 and H4K20me1. Similar to the data on progressive methylation, we found relatively few sites that were converted from H4K20me3 to H4K20me1 (Fig. 6D, E), and analyses of these sites did not reveal any significant pathways. These data suggest that H4K20me1 and H4K20me3 have distinct roles in transcriptional regulation.

## Discussion

Studies examining the role of H4K20 methylation in gene expression have been challenging due to the essential nature of KMT5A (SETD8) and the many complex cellular roles of H4K20 methylation, including cell cycle progression and DNA damage repair. In this study, we examined a well-characterized primary human cell culture model of erythropoiesis to gain insights into the function of H4K20 methylation and its role in regulating gene expression. As has been previously described, we found H4K20me1 and H4K20me3 enrichment in intergenic regions, but we also found enrichment at gene promoters and over gene bodies. Moreover, dynamic changes in these marks that occurred during erythroid maturation mapped primarily to these genic regions. H4K20me1 was enriched over gene bodies and was closely correlated with levels of Pol II and RNA expression, while H4K20me3 enrichment occurred primarily at promoters, where it was associated with an elevated pausing index. Surprisingly, there was little evidence of progressive H4K20 methylation.

Our data suggest a complex relationship between H4K20me1 and Pol II occupancy and activity. We find that H4K20me1 enrichment over the gene body is highly correlated with KMT5A occupancy at the promoter, RNA expression and Pol II occupancy. These data are consistent with studies suggesting that H4K20me1 is co-transcriptionally deposited [18] and facilitates Pol II pause release [18, 41]. They are also consistent with studies suggesting that H4K20me1 over gene bodies is associated with chromatin accessibility [10] and that H4K20me1 enrichment correlates with the speed of Pol II transcription [42]. Intriguingly, several highly expressed genes were enriched for the histone demethylase PHF8, and exhibited a dramatic loss of H4K20me1 during terminal maturation. Enrichment for PHF8 and low levels of H4K20me1 have been observed at highly expressed genes in other cellular systems, such as the albumin gene in hepatocytes [18], although the mechanisms underlying these findings remain elusive.

We also identified subsets of genes that were enriched for H4K20me1 at the transcription start site, and were expressed at low levels at both day 7 and day 10. This correlation suggests that H4K20me1 at the TSS was associated with stable transcriptional repression. Chromatin readers of H4K20me1, such as L3MBTL1, can compact chromatin and repress transcription [15, 43], and deletion of PHF8 is associated with H4K20me1 gain and transcriptional repression [39]. Further, KMT5A and H4K20me1 have been shown to repress specific subsets of genes [44]. Intriguingly, genes that became repressed during maturation from day 7 to day 10 did not accumulate H4K20me1 at the promoter, indicating that this is not a mechanism of transcriptional repression for genes that are dynamically regulated during the end stages of terminal erythropoiesis. Together, these data suggest that the relationship between H4K20me1 and transcription is complex, and that H4K20me1 may attenuate transcription in specific contexts.

**Fig. 7:**
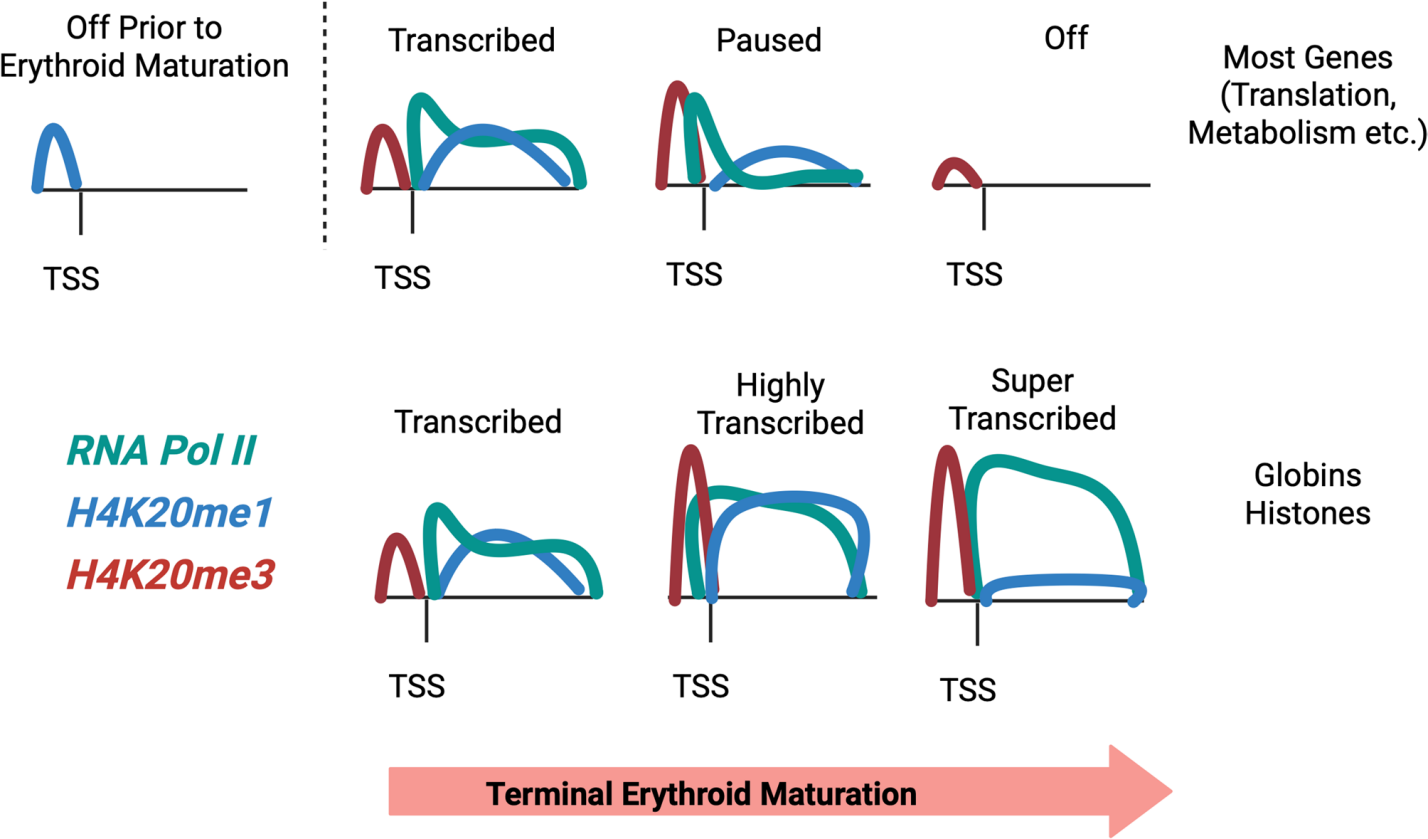
Proposed model depicting the relationship of H4K20 methylation and gene expression

Our data further suggest that H4K20me3 may have a more complex role in gene expression than previously appreciated. We find that the majority of H4K20me3 enrichment is in intergenic regions, where it has a well-defined role in genomic stability and the repression of repetitive elements [16, 45, 46]. We also find H4K20me3 enriched at several highly expressed genes, most notably the globin gene loci and the histone genes, as well as at numerous genes that are dynamically regulated during terminal erythroid maturation. In contrast to its role in the repression of repetitive elements and genome stability, the role of H4K20me3 at transcriptionally active genes is not well understood. In embryonic stem cells, H4K20me3 was noted to be enriched at bivalent promoters, and was associated with genes that lose Pol II occupancy during differentiation [25]. We find here that H4K20me3 marks the promoters of genes that are both activated and repressed in terminally differentiating erythroid cells. We suspect this puzzling duality is due to the fact that promoter H4K20me3 is associated with both actively transcribed genes with high levels of H4K20me1 over the gene body and with “paused” genes with low levels of H4K20me1, with the H4K20me1 status more predictive of the level of transcription (Fig. 7). Together this suggests that although the majority of H4K20me3 is localized outside of genic regions, this modification might play an important role in specific modes of transcriptional regulation. One unexplored mechanism for H4K20 methylation regulation of gene expression is indirect through sequestration of limited chromatin resources. In this way, the intergenic and repetitive elements where the majority of H4K20me3 is localized could potentially serve as methyl group or enzyme reservoirs, limiting histone methylation at genic regions in trans. Similar mechanisms have been recently proposed for histone variant indirect regulation of cellular metabolism genes through a source-sink competition at repetitive elements [47, 48]. 

We and others previously found that KMT5A loss results in considerable upregulation of gene expression [11, 30]. In addition, we identified increased chromatin accessibility at promoters of key erythroid differentiation transcription factors upon KMT5A deletion. These findings naturally lend support to the idea that H4K20me1, the direct enzymatic product of KMT5A, represses transcription. While informative, global KMT5A loss of function (LOF) approaches cannot disentangle (1) the effects of H4K20 monomethylation loss from H4K20 di- or tri-methylation loss, (2) the effects of H4K20 methylation enrichment at different regions of the genome (promoters vs. gene bodies vs. intergenic regions), or (3) secondary effects due to cell cycle disruption or loss of methylation on non-histone KMT5A targets [49–51]. 

Our current understanding of the genomic localization of H4K20me1 and KMT5A supports a nuanced role for KMT5A and H4K20 methylation. Our previous LOF findings are consistent with our current study when we take into consideration dual roles for H4K20me1: enrichment over gene bodies is strongly correlated with RNA Pol II elongation, but enrichment over promoters is associated with low levels of RNA Pol II and corresponding mRNA. Indeed, a dual role for H4K20 methylation at promoters versus gene bodies was purported by Kapoor-Vazirani and Vertino in the context of polymerase pausing regulation in breast cancer cell lines [41]. Similarly, we find that at highly “paused” genes H4K20me1 levels are higher at promoters than gene bodies, while the opposite is true for genes with a low pausing index. We previously found that polymerase pausing in maturing erythroblasts correlates with subsequent Pol II loss and transcriptional downregulation at the majority of genes, while increasingly sparse RNA Pol II is allocated to essential erythroid genes. Here we find gene body H4K20me1 is reduced at the same genes where RNA Pol II is paused and then lost. Taken together, we favor a model in which H4K20me1 promoter methylation promotes RNA Pol II pausing while gene body H4K20me1 is associated with transcriptional elongation (Fig. 7).

Interestingly, we also find that KMT5A overwhelmingly occupies promoters of genes that have high levels of promoter H4K20me3 and PHF8, as well as H4K20me1 enrichment over the gene body. We propose that by early basophilic stages of erythroid maturation, high promoter nucleosome turnover at actively transcribed genes requires vigilant progressive H4K20 methylation as well as H4K20me1 demethylation at promoters to keep H4K20me1 restrained to gene bodies. Thus, the majority of KMT5A protein may be required to maintain adequate H4K20me3 at promoters, with a smaller fraction regulating H4K20me1 at gene bodies. Future studies are needed to better understand the dynamics of KMT5A occupancy over gene bodies at earlier and later stages of erythroid differentiation.

A dynamic interplay of histone methyltransferases and demethylases is required for the establishment and maintenance of H4K20 methylation patterns. While mono-methylation of H4K20 by KMT5A is required for the subsequent generation of both H4K20me2 and H4K20me3, there are several demethylases that can generate H4K20me1 from H4K20me3 and H4K20me2 [46]. In our dataset, H4K20me1 and H4K20me3 occupy distinct regions throughout maturation, with very few regions showing progressive methylation from H4K20me1 to H4K20me3. Of note, however, a limitation of our study is that we have no information on H4K20me2 distribution; the high abundance of this mark throughout the genome and the lack of reliable antibodies make assessing H4K20me2 with genomics approaches extremely difficult. Despite these limitations, these data strongly suggest that in maturing erythroid cells, H4K20me3 has roles in transcriptional regulation beyond repetitive elements and bivalent regions.

## Conclusions

By studying the occupancy of H4K20me1 and H4K20me3 in genic regions in the context of a well-characterized, non-transformed, system of cellular differentiation, we identified dynamic changes in H4K20 methylation during cellular differentiation that correlate strongly with Pol II occupancy and activity. As these marks are essential for normal development, and are frequently dysregulated in cancer, further studies aimed at understanding their roles in regulating transcription are likely to be highly informative for human health and disease.

## Methods

### Cell culture

Human CD34 + cells were provided by the Yale Cooperative Center of Excellence in Hematology, and cultured using a semi synchronous CD36^+^ selection culture system, as previously described [31]. 

### Cytospins

Cytospins of 2 × 103 cells were performed following a wash in PBS followed by a 10-minute incubation in ice in PBS supplemented with 0.01% glucose and 0.03% BSA. Cells were placed on the slide using Cytospin (Shandon Cytospin 2) at 300 RPM for 2 min. The cells were then air dried for 15 min and then fixed in 100% methanol for 5 min. The slide was stained in 1:20 Giemsa (Millipore-Sigma, GS1L) for 30 min and then washed in water. Images were taken at 200x magnification on a Nikon DS-Fi1 camera using NIS elements software (Nikon).

### Biochemical analysis

Protein extracts from cultured cells were collected in RIPA buffer (Cell Signaling Technology) and sonicated for 30 s on and 30 s off for a total of 10 cycles in a Biorupter (Diagenode). Lysates were then resolved, transferred and blotted using antibodies for Total H4 (CS 2935), H4K20me1 (Millipore 07-1570) and H4K20me3 (abcam ab9053). Blots imaged using C-DiGit Blot Scanner (Li-Cor) after five-minute exposure to WesternSure PREMIUM Chemilunescent Substrate (Li-Cor). Mass spectrometry data generated previously; methods described in Murphy 2021^32^.

### Flow cytometry

Cultured cells were analyzed for immunophenotype following staining as previously described [52] with antibodies for CD36-FITC (clone CB38, BD Biosciences), CD235a-PECy7 (clone GA-R2, BD Biosciences), CD49d-PE (clone MZ18-24A9, Miltenyi Biotec), and BAND3 (Bric 200, IBGRL). BAND3 surface protein expression was analyzed using a secondary antibody (anti-IgG APC-Cy7, clone MOPC-21, BD Biosciences). Additionally, DAPI (ThermoFisher Scientific, D1306) and DRAQ5 (ThermoFisher Scientific, 65-0880-92) were added to identify live cells and nuclei, respectively. Cells were run either on an Image Stream X (Amnis/EMD Millipore) and analyzed with IDEAS 6.3 (Amnis/EMD Millipore); or an LSRII (BD Biosciences) and analyzed with FCS Express (DeNovo Software, v7). Cytoplasmic and nuclear area were determined as previously described [53]. 

### CUT&Tag/CUT&RUN

Early basophilic erythroblasts or orthochromatic erythroblasts were collected for CUT&Tag (approximately 100,000 cells per replicate) and CUT&RUN (approximately 500 000 cells per replicate). CUT&Tag was performed in triplicate for each antibody following the Bench top V.1 protocol [34]. Briefly, cells were bound to Concanavalin A beads, and incubated in primary antibodies overnight at 4 degrees, secondary antibodies for 1 h at RT, and pA-Tn5 adapter complex for 1 h at RT. Tagmentation was induced for 1 h, and DNA was extracted with phenol-chloroform-isomyl alcohol. Libraries were amplified using NEBNext HiFi 2X Master mix, pooled, and cleaned up using 1.3X vol. SPRI beads. H4K20me1 Millipore (cat# 07-1570) antibody was used for CUT&Tag.

CUT&RUN assays were performed in triplicate using the Epicypher CUTANA CUT&RUN protocol v1.6. Libraries were generated using the NEBNext Ultra II DNA Library Prep kit (NEB). Antibodies H4K20me3 abcam (cat# ab9053),PHF8 abcam (cat# ab36068), H4K20me1 Millipore (cat# 07-1570), and KMT5A abcam (cat# ab3798) were used for CUT&RUN. For PHF8 and KMT5A, TF specific size selection was optimized for small fragments (end prep for 20 C for 30 min followed by 50 C for 60 min, ligation AMPure XP cleanup at 1.75X, annealing temperature of 65 C for library amplification, and library AMPure XP cleanup using 1st round negative selection at 0.8X followed by a 2nd round positive selection at 1.2X). Libraries were sequenced as 150 bp paired end using the Illumina HiSeq platform.

### Data processing

Previously published data analyzed in this study were re-processed from raw Fastq format. All datasets used are listed in supplemental Table 1. CUT&Tag, CUT&RUN, and ChIP-seq Fastq files were aligned to the hg38 reference genome using Bowtie2. For CUT&Tag and CUT&RUN, PCR duplicates were removed using Picard. RNA-seq Fastq files were aligned to the hg38 reference genome using STAR, RPKM normalized using bamCoverage. RPKM read count normalization was performed on alignment files using deepTools [54] bamCoverage, and replicate merged bigwig files were generated using deepTools bigWigMerge with adjust = 1.0.

### Bioinformatics

Peaks were called using macs2 [55] bdgpeakcall with -c parameter set to 450 (H4K20me1), 200 (H4K20me3), or 150 (PHF8). Peak annotation was performed using Homer annotatePeaks.pl. Heatmaps and profile plots were generated using deepTools. Region-matched enrichment scores over gene promoters (TSS +/- 500 bp), gene bodies (TSS to TES), or merged exons (metagene option for RNA-seq) were generated in deepTools. Parameters --outFileNameMatrix and --outFileSortedRegions were used to export tables. Pearson correlations were calculated in R. Correlation heatmaps were generated using pheatmap in R. Deciles of H4K20me1 (average score over gene body) or RNA (average score over merged exons) were calculated using ranked scores in R, and region files were subset and exported for generation of profile plots using deepTools. Pausing index proxy was calculated using average promoter Ser5-PolII (TSS +/- 500 bp) / gene body (TSS to TES). PI > 4 and PI < 4 subsets were generated for expressed genes (D7 average RNA RPKM > 100) in R. Welch Two Sample t-test was used for statistics in R. Gene ontology and pathway analysis was performed using the Enrichr database.

Differential analysis on peaks was performed using DiffBind (R) on pre-normalized bam files. DESeq2 was used for differential analysis over gene body or promoter regions using read count tables over the entire gene length (TSS to TES). Count tables for individual replicates were generated using average enrichment scores over gene bodies (H4K20me1) or promoters (H4K20me3) (genes with minimum threshold RPKM > 25 were used for DESeq2). Volcano plots were generated using ggplot in R. Region files for differential genes with adj. *p* value < 0.05 were subset, exported and used for heatmap and profile plot generation in deepTools. Boxplots for H4K20me1 and H4K20me3 differential genes were generated using enrichment scores over gene promoters (TSS +/- 500 bp) or gene bodies (TSS to TES) from deepTools, and plotted in R.

Region-matched enrichment scores over gene promoters (TSS +/- 500 bp), gene bodies (TSS to TES), or merged exons (metagene option for RNA-seq) were generated in deepTools. Parameters --outFileNameMatrix and --outFileSortedRegions were used to export tables. Pearson correlations were calculated in R. Correlation heatmaps were generated using pheatmap in R. Density correlation plots were generated in R using ggplot for genes with average scores > 0 for each axis. r represents pearson correlation coefficient. Heatmaps and profile plots were generated using deepTools. Deciles of H4K20me1 (average score over gene body) or RNA (average score over merged exons) were calculated using ranked scores in R, and region files were subset and exported for generation of profile plots using deepTools. L2FC quantiles were calculated using ranked scores in R using minimum expression threshold (average RNA RPKM > 1).

## Electronic supplementary material

Below is the link to the electronic supplementary material.

Supplementary Material 1

## Data Availability

Data generated in this study are available under Geo Superseries accession GSE260722, comprised of subseries GSE260720 (CUT&RUN) and GSE260721 (CUT&TAG).
