# Supplementary material for: Histone H4 lysine 20 methylation marks genes dynamically regulated during erythroid maturation
Source: Epigenetics Chromatin. 2025 Jul 26;18:48. doi: 10.1186/s13072-025-00609-2 (PMC12296644; doi:10.1186/s13072-025-00609-2)
Supplement: Supplementary file 1 — Supplementary Material 1 [file 13072_2025_609_MOESM1_ESM.pdf]

Sup. Fig. 1

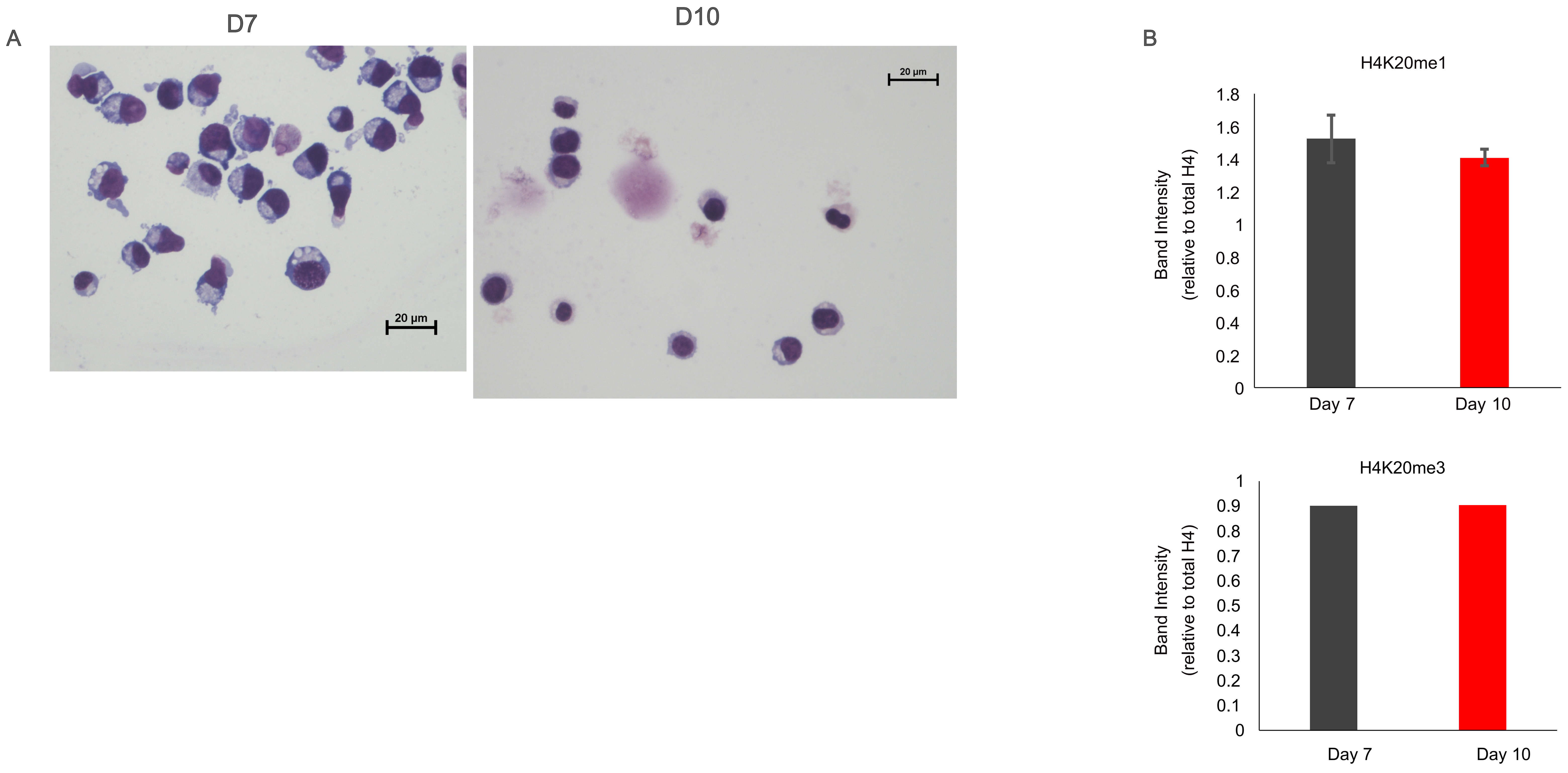
A
D7
D10
20 µm
20 µm
B
H4K20me1
Band Intensity
(relative to total H4)
1.8
1.6
1.4
1.2
1
0.8
0.6
0.4
0.2
0
Day 7
Day 10
H4K20me3
Band Intensity
(relative to total H4)
1
0.9
0.8
0.7
0.6
0.5
0.4
0.3
0.2
0.1
0
Day 7
Day 10

Sup. Fig. 2

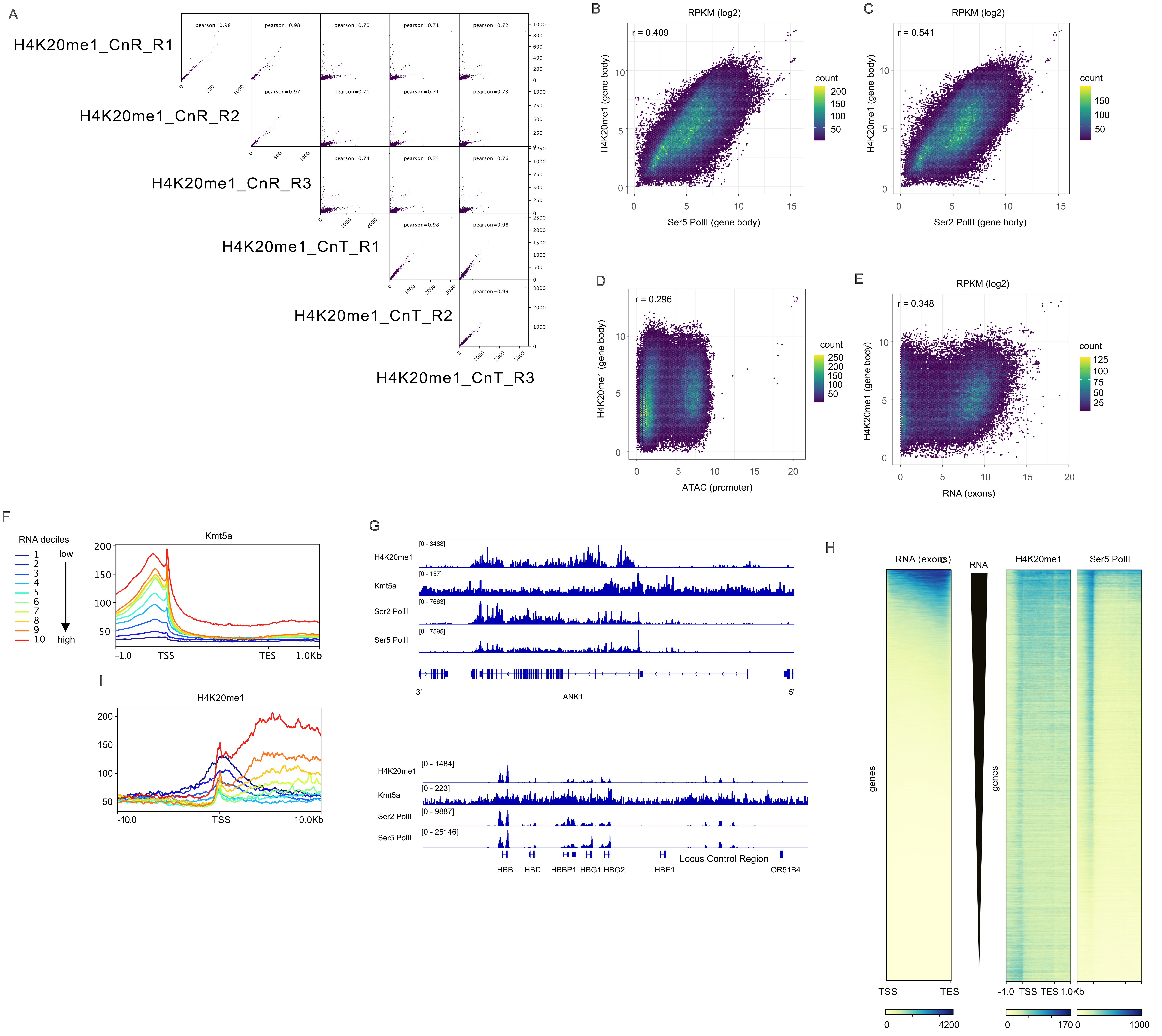

A
H4K20me1_CnR_R1
H4K20me1_CnR_R2
H4K20me1_CnR_R3
H4K20me1_CnT_R1
H4K20me1_CnT_R2
H4K20me1_CnT_R3
B
RPKM (log2)
r = 0.409
H4K20me1 (gene body)
Ser5 PolII (gene body)
count
C
RPKM (log2)
r = 0.541
H4K20me1 (gene body)
Ser2 PolII (gene body)
count
D
r = 0.296
H4K20me1 (gene body)
ATAC (promoter)
count
E
RPKM (log2)
r = 0.348
H4K20me1 (gene body)
RNA (exons)
count
F
RNA deciles
low
high
Kmt5a
-1.0
TSS
TES
1.0Kb
I
H4K20me1
-10.0
TSS
10.0Kb
G
H4K20me1
Kmt5a
Ser2 PolII
Ser5 PolII
3'
ANK1
5'
Locus Control Region
HBB
HBD
HBBP1
HBG1
HBG2
HBE1
OR51B4
H
RNA (exons)
RNA
H4K20me1
Ser5 PolII
genes
TSS
TES
-1.0 TSS TES 1.0Kb

Sup Fig 3

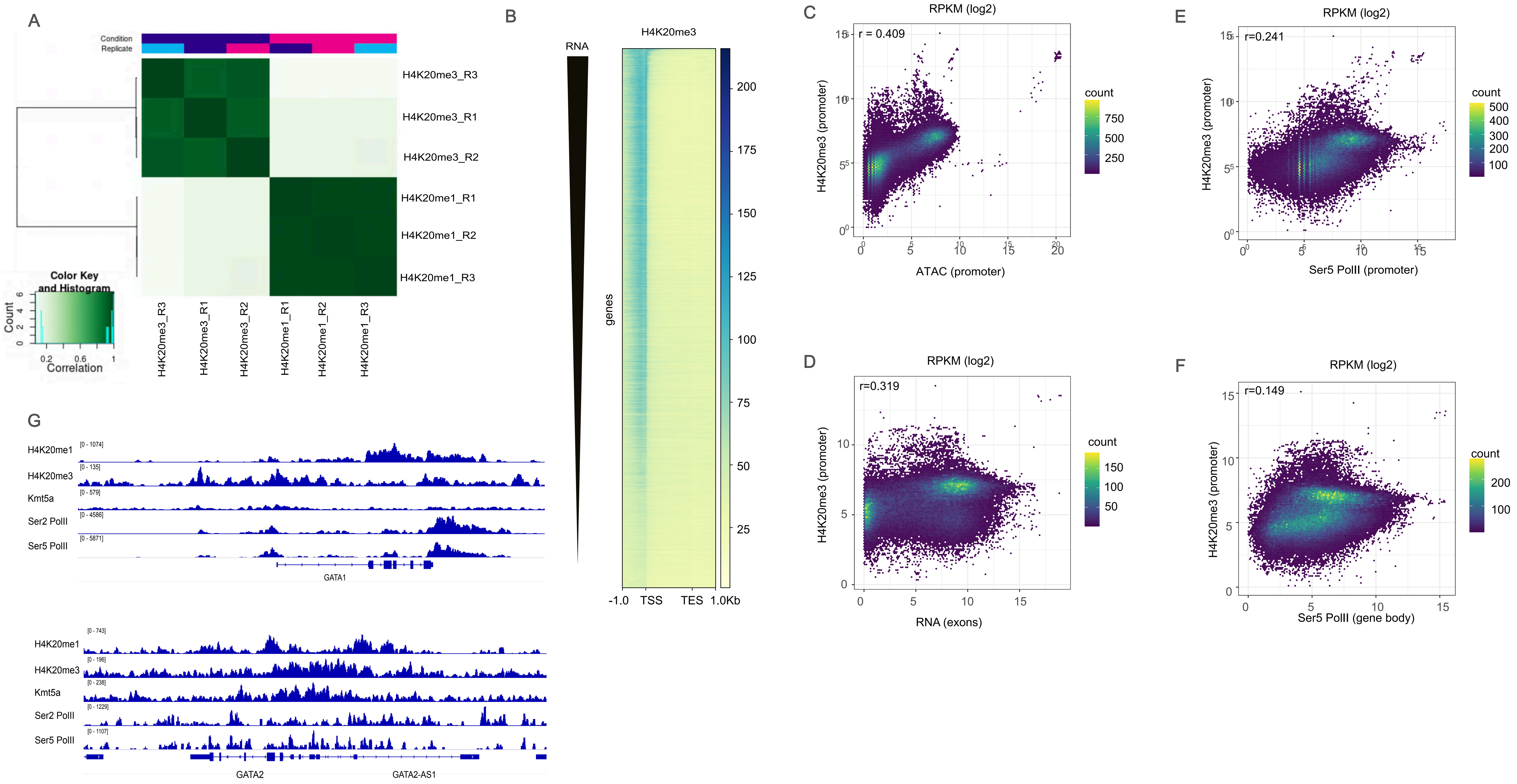

A
Condition
Replicate
H4K20me3_R3
H4K20me3_R1
H4K20me3_R2
H4K20me1_R1
H4K20me1_R2
H4K20me1_R3
Color Key and Histogram
Count
Correlation
B
RNA
H4K20me3
genes
-1.0 TSS TES 1.0Kb
C
RPKM (log2)
r = 0.409
H4K20me3 (promoter)
ATAC (promoter)
count
D
RPKM (log2)
r=0.319
H4K20me3 (promoter)
RNA (exons)
count
E
RPKM (log2)
r=0.241
H4K20me3 (promoter)
Ser5 PolII (promoter)
count
F
RPKM (log2)
r=0.149
H4K20me3 (promoter)
Ser5 PolII (gene body)
count
G
H4K20me1
H4K20me3
Kmt5a
Ser2 PolII
Ser5 PolII
GATA1
H4K20me1
H4K20me3
Kmt5a
Ser2 PolII
Ser5 PolII
GATA2
GATA2-AS1

Sup. Figure 4

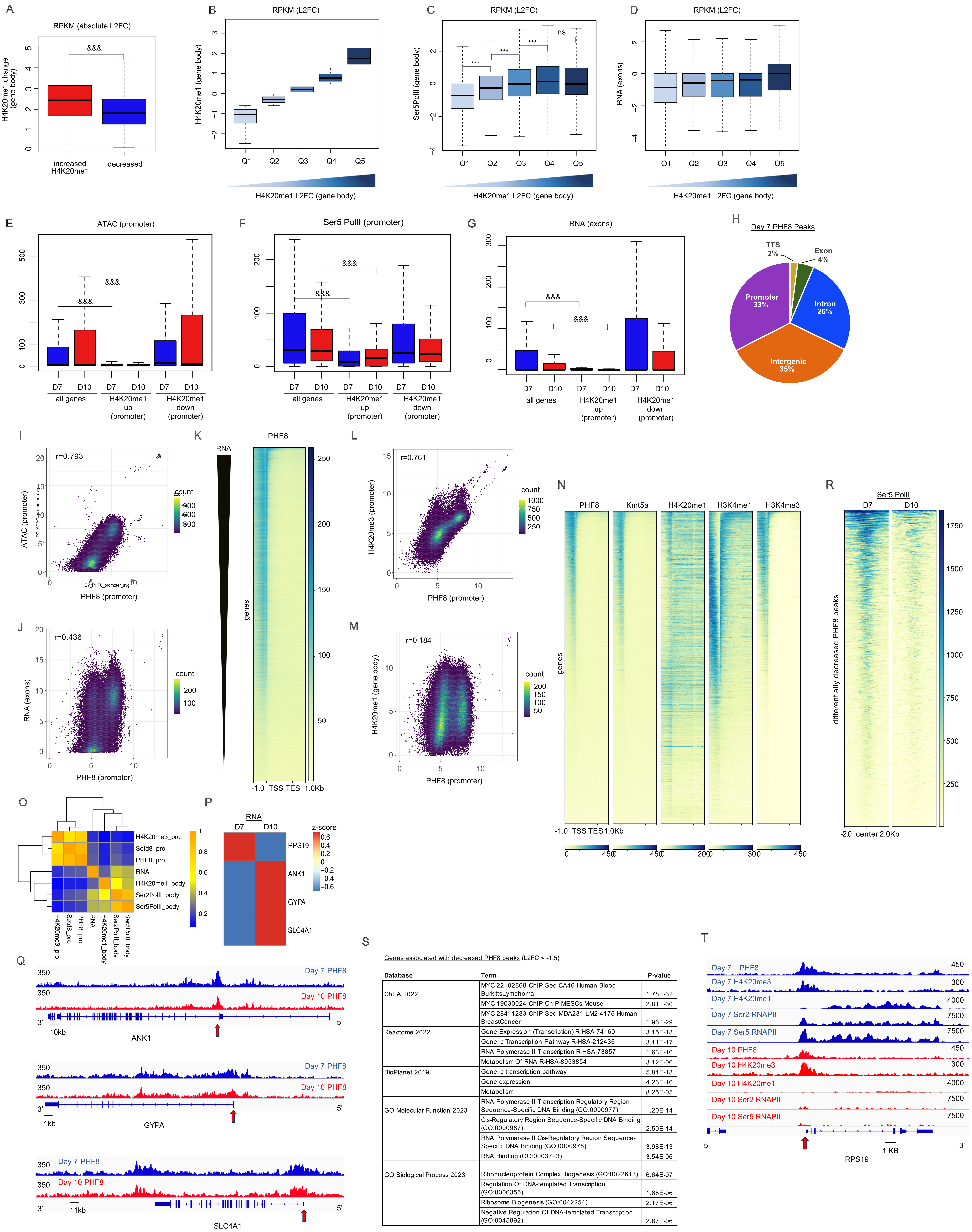

Genes associated with decreased PHF8 peaks (L2FC < -1.5)

Database	Term	P-value
ChEA 2022	MYC 22102868 ChIP-Seq CA46 Human Blood BurkittsLymphoma	1.78E-32
	MYC 19030024 ChIP-ChIP MESCs Mouse	2.81E-30
	MYC 28411283 ChIP-Seq MDA231-LM2-4175 Human BreastCancer	1.96E-29
Reactome 2022	Gene Expression (Transcription) R-HSA-74160	3.15E-18
	Generic Transcription Pathway R-HSA-212436	3.11E-17
	RNA Polymerase II Transcription R-HSA-73857	1.63E-16
	Metabolism Of RNA R-HSA-8953854	3.12E-06
BioPlanet 2019	Generic transcription pathway	5.84E-18
	Gene expression	4.26E-16
	Metabolism	8.25E-05
GO Molecular Function 2023	RNA Polymerase II Transcription Regulatory Region Sequence-Specific DNA Binding (GO:0000977)	1.20E-14
	Cis-Regulatory Region Sequence-Specific DNA Binding (GO:0000987)	2.50E-14
	RNA Polymerase II Cis-Regulatory Region Sequence-Specific DNA Binding (GO:0000978)	3.98E-13
	RNA Binding (GO:0003723)	3.54E-06
GO Biological Process 2023	Ribonucleoprotein Complex Biogenesis (GO:0022613)	6.64E-07
	Regulation Of DNA-templated Transcription (GO:0006355)	1.68E-06
	Ribosome Biogenesis (GO:0042254)	2.17E-06
	Negative Regulation Of DNA-templated Transcription (GO:0045892)	2.87E-06

Sup. Fig 5

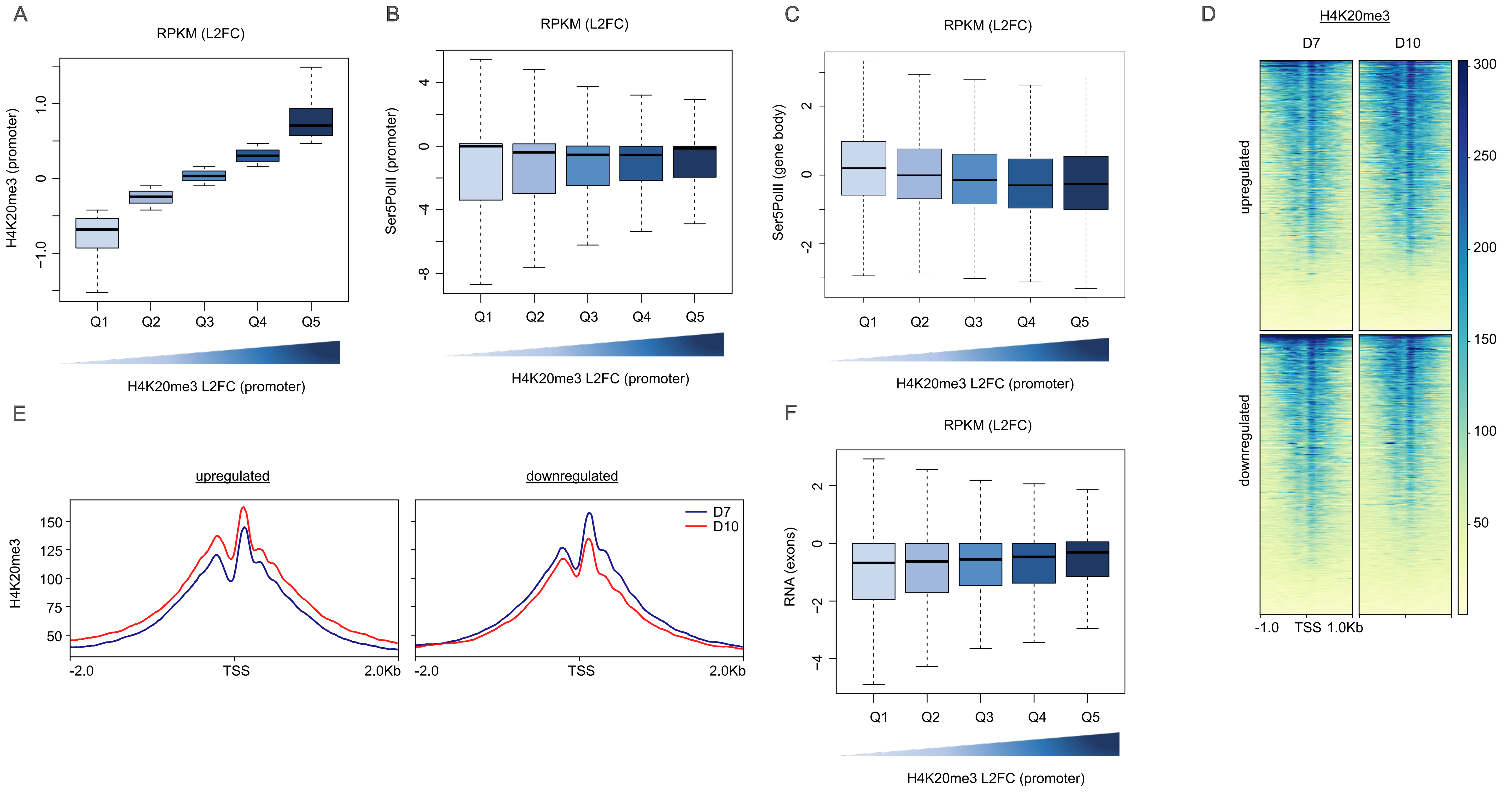
A
RPKM (L2FC)
H4K20me3 (promoter)
Q1
Q2
Q3
Q4
Q5
H4K20me3 L2FC (promoter)
B
RPKM (L2FC)
Ser5PolII (promoter)
H4K20me3 L2FC (promoter)
C
RPKM (L2FC)
Ser5PolII (gene body)
H4K20me3 L2FC (promoter)
D
H4K20me3
D7
D10
upregulated
downregulated
-1.0
TSS
1.0Kb
300
250
200
150
100
50
E
upregulated
downregulated
H4K20me3
D7
D10
-2.0
TSS
2.0Kb
F
RPKM (L2FC)
RNA (exons)
H4K20me3 L2FC (promoter)

Sup Fig. 6

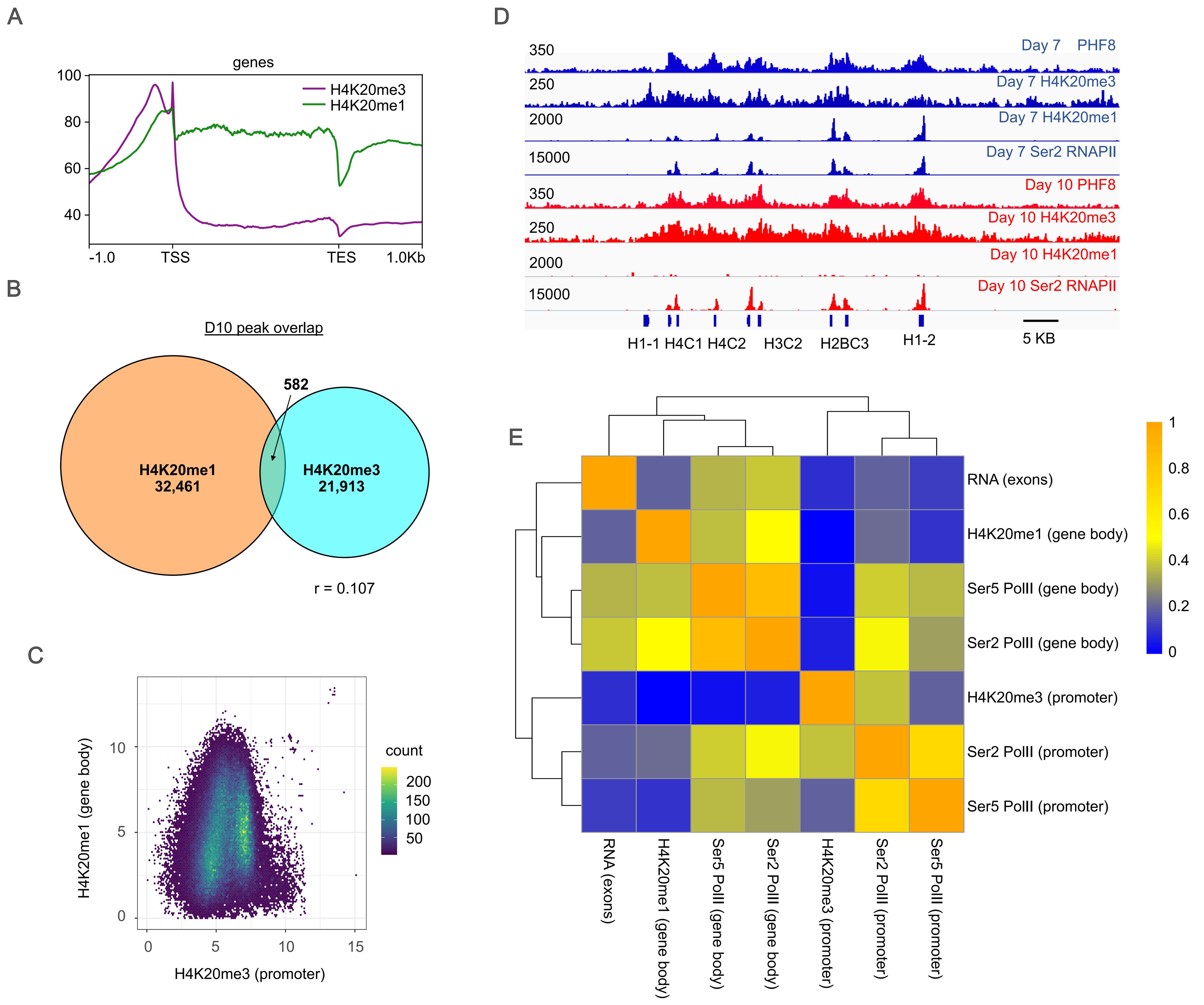

A
genes
H4K20me3
H4K20me1
100
80
60
40
-1.0
TSS
TES
1.0Kb
B
D10 peak overlap
582
H4K20me1
32,461
H4K20me3
21,913
r = 0.107
C
H4K20me1 (gene body)
H4K20me3 (promoter)
count
200
150
100
50
D
Day 7 PHF8
Day 7 H4K20me3
Day 7 H4K20me1
Day 7 Ser2 RNAPII
Day 10 PHF8
Day 10 H4K20me3
Day 10 H4K20me1
Day 10 Ser2 RNAPII
350
250
2000
15000
H1-1 H4C1 H4C2 H3C2 H2BC3 H1-2
5 KB
E
RNA (exons)
H4K20me1 (gene body)
Ser5 PolII (gene body)
Ser2 PolII (gene body)
H4K20me3 (promoter)
Ser2 PolII (promoter)
Ser5 PolII (promoter)
1
0.8
0.6
0.4
0.2
0

Supplmental Table 1. Genomics datasets analyzed as part of this study.

Target	Citation
H4K20me1	generated for this study
H4K20me3	generated for this study
PHF8	generated for this study
KMT5A	generated for this study
H4K20me1	generated for this study
mRNA	Murphy ZC, Murphy K, Myers J, Getman M et al. Regulation of RNA polymerase II activity is essential for terminal erythroid maturation. Blood 2021 Nov 4;138(18):1740-1756. PMID: 34075391
Ser5 Pol II	Murphy ZC, Murphy K, Myers J, Getman M et al. Regulation of RNA polymerase II activity is essential for terminal erythroid maturation. Blood 2021 Nov 4;138(18):1740-1756. PMID: 34075391
Ser2 Pol II	Murphy ZC, Murphy K, Myers J, Getman M et al. Regulation of RNA polymerase II activity is essential for terminal erythroid maturation. Blood 2021 Nov 4;138(18):1740-1756. PMID: 34075391
H3K36me3	Murphy ZC, Murphy K, Myers J, Getman M et al. Regulation of RNA polymerase II activity is essential for terminal erythroid maturation. Blood 2021 Nov 4;138(18):1740-1756. PMID: 34075391
chromatin accessibility	Schulz VP, Yan H, Lezon-Geyda K, An X et al. A Unique Epigenomic Landscape Defines Human Erythropoiesis. Cell Rep 2019 Sep 10;28(11):2996-3009.e7. PMID: 31509757
H3K4me1	Huang J, Liu X, Li D, Shao Z et al. Dynamic Control of Enhancer Repertoires Drives Lineage and Stage-Specific Transcription during Hematopoiesis. Dev Cell2016 Jan 11;36(1):9-23. PMID: 26766440
H3K4me3	Huang J, Liu X, Li D, Shao Z et al. Dynamic Control of Enhancer Repertoires Drives Lineage and Stage-Specific Transcription during Hematopoiesis. Dev Cell2016 Jan 11;36(1):9-23. PMID: 26766440

GEO accession	Technique	Antibody
GSE260721	CUT&Tag	H4K20me1 Millipore (cat# 07-1570)
GSE260720	CUT&RUN	abcam (cat# ab9053)
GSE260720	CUT&RUN	abcam (cat# ab36068)
GSE300158	CUT&RUN	abcam (Cat# ab3798)
GSE300158	CUT&RUN	H4K20me1 Millipore (cat# 07-1570)
GSE155848	RNA-seq	N/A
GSE171492	CUT&Tag	see publication methods
GSE171492	CUT&Tag	see publication methods
GSE155847	ChIP-seq	see publication methods
GSE128266	ATAC-seq	N/A
GSE70660	ChIP-seq	see publication methods
GSE70660	ChIP-seq	see publication methods

Supplemental Figure legends

Fig S1. (A) Additional day 7 and Day 10 cell morphology cytospin images. (B) Quantification of western blots shown in Figure 1.

Fig S2. (A) Correlation scatter plot of Day 7 CUT&RUN and CUT&Tag for H4K20me1. (B-E) Density plots of average enrichment scores comparing (B) H4K20me1 over gene bodies (TSS-TES) vs. Ser5 Pol II over gene bodies, (C) H4K20me1 over gene bodies (TSS-TES) vs. Ser2 Pol II over gene bodies, (D) H4K20me1 over gene bodies (TSS-TES) vs. ATAC over promoters (TSS+/- 500bp), (E) H4K20me1 over gene bodies (TSS-TES) vs. RNA over merged exons (metagene). r value represents pearson correlation coefficient. (F) Profile plot of H4K20me1 plotted over genes divided into deciles based on ranked RNA levels (merged exons), centered on the TSS +/- 10kb. (G) Gene tracks showing example of H4K20me1, KMT5A, ser 2 Pol II, and ser 5 pol II at the ANK gene and Beta globin locus. Y axis represents RPKM. (H) Heat map of RNA (merged exons) plotted over gene transcripts, sorted by RNA, for which the sort order was preserved for heat map of H4K20me1 and Ser5 Pol II.

Fig S3. (A) Pearson correlation heatmap for average normalized scores of H4K20me3 and H4K20me1. (B) Heat map of H4K20me3 over genes sorted by average RNA (merged exons). (C-F) Density plots of average enrichment scores comparing (C) H4K20me3 over gene promoters (TSS+/- 500bp) vs. ATAC over promoters (TSS+/- 500bp), (D) H4K20me3 over promoters (TSS+/- 500bp) vs. RNA over merged exons (metagene). (E) H4K20me3 over gene promoters (TSS+/-

500bp) vs. Ser5 Pol II over promoters (TSS+/- 500bp), (F) H4K20me3 over promoters (TSS+/- 500bp) vs. Ser5 Pol II over gene bodies. (G) Gene tracks showing example of H4K20me1, H4K20me3, Kmt5a, ser2 Pol II, and ser5 Pol II enrichment at day 7, y axis represents RPKM.

Fig S4. (A) Boxplot showing average absolute RPKM L2FC of peaks with differentially increased H4K20me1 (red) vs decreased H4K20me1 (blue). &&& indicates significantly decreased p value < 2.2e-16. (B) boxplot showing H4K20me1 L2FC RPKM in 5 equal quartiles of genes based on L2FC H4K20me1 over gene bodies (TSS to TES) day 10 vs. day 7. (C-D) Boxplot of (C) Ser5 Pol II L2FC over gene bodies (TSS to TES), and (D) RNA L2FC (merged exons, metagene) in same H4K20me1 L2FC gene quartiles as (B) *** indicates significantly increased p value < 2.2e-16. (E-G) Boxplot showing average (E) ATAC over promoters (TSS +/- 500bp), (F) Ser5 PolII over promoters (TSS +/- 500bp), and (G) RNA over merged exons (metagene) at day 7 (blue) and day 10 (red) over all genes, subset of genes for which average H4K20me1 increases over promoters (TSS +/- 500bp > 4 (10,553), and subset of genes for which average decreases H4K20me1 over promoters (TSS +/- 500bp < 4 (14,297). &&& indicates significantly decreased p value < 2.2e-16. (H) Genomic annotation of PHF8 peaks (22,845 total) on day 7 of maturation. (I-J) Density plots of average enrichment scores comparing (I) PHF8 over gene promoters (TSS+/- 500bp) vs. ATAC over promoters (TSS+/- 500bp), and (J) PHF8 over gene promoters (TSS+/- 500bp) vs. RNA over merged exons (metagene). (K) Heat map of PHF8 over genes sorted by average RNA (merged exons). (L-M) Density plots of average enrichment scores comparing (L) PHF8 over gene promoters (TSS+/- 500bp) vs. H4K20me3 over promoters (TSS+/- 500bp), and (M) PHF8 over gene promoters (TSS+/- 500bp) vs. H4K20me1 over gene bodies (TSS-TES). r-value represents pearson correlation

coefficient. (N) Heat map showing PHF8, H4K20me1, H3K4me1 and H3K4me3 occupancy over genes sorted by PHF8 levels on day 7. (O) Pearson correlation heatmap of indicated factors. (P) Heatmap demonstrating RNA expression of indicated genes at day 7 and day 10. (Q) Gene tracks showing example of PHF8 enrichment at day 7 (blue) and day 10 (red) over the ANK1, GYPA, and SLC4A1 genes, y axis represents RPKM, genome scale bar as indicated. (R) Heat map showing serine 5 phosphorylated RNA Polymerase II (Ser5 Pol II) over differentially decreased PHF8 peaks at day 7 and day 10. (S) Gene ontology and pathway analysis for genes associated with differentially decreased PHF8 peaks. (T) Additional gene tracks showing example of PHF8, H4K20me3, H4K20me1, Ser5 Pol II, and Ser2 Pol II enrichment at day 7 (blue) and day 10 (red) over the RPS19 gene, y axis represents RPKM, genome scale bar as indicated.

Fig S5. (A) boxplot showing H4K20me3 L2FC RPKM in 5 equal quartiles of genes based on L2FC H4K20me3 over promoters (TSS +/- 500bp) day 10 vs. day 7. (B-C) Boxplot of Ser5 Pol II L2FC over (B) promoters (TSS +/- 500bp), and (C) gene bodies (TSS t TES) in same H4K20me3 L2FC gene quartiles as (A). (D) Heat map and (E) profile plot showing H4K20me3 plotted over upregulated and downregulated genes at day 7 and day 10. (F)RNA L2FC (merged exons, metagene) L2FC, in same H4K20me3 L2FC gene quartiles as (A).

Fig. S6. (A) Profile plot of H4K20me1 (green) and H4K20me3 (purple) over genes. (B) Venn diagram showing direct overlap of day 10 H4K20me1 and H4K20me3 peaks. (C) Density plots of average enrichment scores comparing H4K20me3 over gene promoters (TSS+/- 500bp) vs.

H4K20me1 over gene bodies (TSS to TES). (D) Additional gene tracks showing example of PHF8, H4K20me3, H4K20me1, and Ser2 PolII enrichment at day 7 (blue) and day 10 (red) over the histone 1 cluster locus. (E) Day 7 Pearson correlation heatmap for average normalized scores of H4K20me1, H4K20me3, Ser5 PolII, Ser2 PolII, and RNA (RNA-seq) over promoters (TSS +/- 500bp), gene bodies (TSS to TES), or merged exons for RNA (metagene) as indicated.
